# Genome-Wide Identification and Functional Analysis of NADPH Oxidase Family Genes in Wheat During Development and Environmental Stress Responses

**DOI:** 10.3389/fpls.2018.00906

**Published:** 2018-07-23

**Authors:** Chun-Hong Hu, Xiao-Yong Wei, Bo Yuan, Lin-Bo Yao, Tian-Tian Ma, Peng-Peng Zhang, Xiang Wang, Peng-Qi Wang, Wen-Ting Liu, Wen-Qiang Li, Lai-Sheng Meng, Kun-Ming Chen

**Affiliations:** ^1^State Key Laboratory of Crop Stress Biology in Arid Areas, College of Life Sciences, Northwest A&F University, Yangling, China; ^2^Department of General Biology, College of Life Science and Agronomy, Zhoukou Normal University, Zhoukou, China; ^3^The Key Laboratory of Biotechnology for Medicinal Plant of Jiangsu Province, School of Life Sciences, Jiangsu Normal University, Xuzhou, China

**Keywords:** wheat (*Triticum aestivum*), NADPH oxidases, phylogenetic analysis, abiotic/biotic stress response, coexpression analysis

## Abstract

As the key producers of reactive oxygen species (ROS), NADPH oxidases (NOXs), also known as respiratory burst oxidase homologs (RBOHs), play crucial roles in various biological processes in plants with considerable evolutionary selection and functional diversity in the entire terrestrial plant kingdom. However, only limited resources are available on the phylogenesis and functions of this gene family in wheat. Here, a total of 46 NOX family genes were identified in the wheat genome, and these NOXs could be classified into three subgroups: typical TaNOXs, TaNOX-likes, and ferric reduction oxidases (TaFROs). Phylogenetic analysis indicated that the typical TaNOXs might originate from TaFROs during evolution, and the TaFROs located on Chr 2 might be the most ancient forms of TaNOXs. TaNOXs are highly expressed in wheat with distinct tissue or organ-specificity and stress-inducible diversity. A large-scale expression and/or coexpression analysis demonstrated that TaNOXs can be divided into four functional groups with different expression patterns under a broad range of environmental stresses. Different TaNOXs are coexpressed with different sets of other genes, which widely participate in several important intracellular processes such as cell wall biosynthesis, defence response, and signal transduction, suggesting their vital but diversity of roles in plant growth regulation and stress responses of wheat.

## Introduction

Reactive oxygen species (ROS) have dual roles in plant growth regulation and environmental stress response. They not only lead to programmed cell death by damaging the cellular components as deleterious factors of oxidative metabolism but are also necessary for plant tolerance to different biotic and abiotic stresses acting as signal molecules (Suzuki et al., 2011; Dietz et al., 2016). Although there are various ROS-generating pathways in plants, the plasma membrane NADPH oxidases (NOXs), also named after respiratory burst oxidase homologs (RBOHs), were considered to be the key enzymes for apoplastic ROS production under both normal and stress conditions and play crucial roles in various biological processes in plants.

NOX homologs are universally present in a broad range of organisms including animals, higher plants, and fungi (Bedard et al., 2007). Until recently, various types of NOXs including the ancestral NOXs, namely ferric reductases (FREs) or ferric reduction oxidases (FROs), were reported in life kingdom with four types. These types were NOXA, NOXB, NOXC, and FRE in fungi (Aguirre et al., 2005). There were also seven types including NOX1–5, DUOX1, and DUOX2 in animals (Bedard et al., 2007) and one type in plants known as NOX5-Like (Sagi and Fluhr, 2006). Although the first NOX gene *OsRbohA* (*OsNOX2*) was characterized as a gp91^phox^ homolog of mammalian NOX in rice, multiple NOX members were identified in plants such as *Arabidopsis thaliana* (Sagi and Fluhr, 2006), rice (Wang et al., 2016), maize (Potocký et al., 2007), tobacco (Yoshioka et al., 2001), potato (Yoshioka et al., 2003), tomato (Amicucci et al., 1999), *Medicago truncatula* (Marino et al., 2011), and *Phaseolus vulgaris* (Nestler et al., 2014), and more than 50 FRO and 77 NOX gene homologs were identified (Chang et al., 2016). All the plant NOX members are NOX5-like homologs except the ancestral NOXs (FROs), which were considered as the isoforms of yeast FREs (Sagi and Fluhr, 2006; Bedard et al., 2007). In addition to having their special NADPH_Ox domain and several calcium-binding EF-hand motifs in its N terminus, the NOX5-like homologs in plants also contain membrane-spanning domains, two hemes, NADPH-binding motifs, and FAD-binding motifs just like all the other NOX/DUOX enzymes in animals (Geiszt, 2006). Many plants have multiple NOX members, and each member has its own special function during the growth and development regulation and stress response. In *Arabidopsis*, there are 10 typical NOX members reported as AtRbohA–J (Sagi and Fluhr, 2006). Among these NOXs, AtRbohD was found to participate in various important processes including abscisic acid-mediated ROS production and stomatal closure (Zhang et al., 2009), lignin assembly (Denness et al., 2011), endosperm development (Penfield et al., 2006) as well as in systemic signal transduction, (Miller et al., 2009), and survival of seedlings under anoxic conditions (Yamauchi et al., 2017). Moreover, it was reported that AtRbohB mainly participates in seed after ripening (Muller et al., 2009), AtRbohC regulates root hair formation and root-hair-cell growth (Takeda et al., 2008), whereas both AtRbohD and AtRbohF not only regulate the immune response (Chaouch et al., 2012) and salt stress tolerance (Xie et al., 2011) but also function in jasmonic acid (JA)-induced gene expression (Maruta et al., 2011). Furthermore, AtRbohE was found to regulate tapetal programmed cell death (PCD) and pollen development (Xie et al., 2011), whereas AtRbohH and AtRbohJ function in pollen tube development (Kaya et al., 2014). In rice, at least nine typical NOX members were identified to be of OsRbohA–I or OsNOX1–9 (Wong et al., 2007; Wang et al., 2013). It was found that OsRbohA (OsNOX2), OsRbohB (OsNOX1), and OsRbohE (OsNOX3) are involved in immune response (Yoshie et al., 2005; Nagano et al., 2016), and OsRbohA (OsNOX2) also plays a crucial role in drought-stress tolerance (Wang et al., 2016). Most recently, OsRbohH (OsNOX9) was found to regulate the aerenchyma formation in roots of rice (Yamauchi et al., 2017). In maize, both ZmRbohH (ZmNOX13) and ROOTHAIRLESS5 (ZmNOX7) were found to function in root development (Rajhi et al., 2011; Nestler et al., 2014).

Moreover, increasing evidence shows that NOXs in plants may serve as molecular “hubs” during ROS-mediated signal transduction pathways (Marino et al., 2012), including receptor-activated C-kinases (RACKs) (Nakashima et al., 2008), Ca^2+^-dependent protein kinases (CDPKs) (Dubiella et al., 2013), mitogen-activated protein kinases (MAPKs) (Asai et al., 2008), phospholipase Dα1 and phosphatidic acid (Zhang et al., 2009), Ca^2+^ (Wudick and Feijo, 2014), phosphatidylinositol (Kaye et al., 2011), NO (Delledonne et al., 2002), cGMP (Li et al., 2011), and extracellular ATP-mediated signaling pathways (Song et al., 2006), and hormonal signaling networks (Mittler et al., 2011). Furthermore, AtRbohD was found to participate in the clathrin- and membrane microdomain-dependent endocytic pathways in *Arabidopsis* (Hao et al., 2014). Although previous studies have highlighted the biochemical properties and physiological functions of NOXs to a certain degree, considering the multiple members in different plant species in which only a few of NOXs were studied in detail, the functions of NOX family proteins are still under investigation.

Wheat (*Triticum aestivum*) is a worldwide staple crop, necessitating a clear deciphering of its developmental characteristics and stress tolerance mechanisms. However, as of today, only two NOXs in wheat have been reported to participate in brown rust infection response (Dmochowska-Boguta et al., 2013), and the functions of wheat NOX family genes and their regulatory mechanisms in both plant growth regulation and environmental stress response are still largely unknown. In the present study, a comprehensive analysis based on bioinformatics approaches and experimental methods was performed to identify the wheat NOX family genes and their functions during the plant development and stress response. The results obtained here will largely broaden our understanding of the roles of NOXs and their regulation in plants, especially in wheat.

## Materials and methods

### Sequence retrieval and identification of the NOX gene family in wheat

We retrieved the potential sequences of NOX family members in wheat from IWGSC (http://www.wheatgenome.org/, last accessed May 25, 2017), NCBI (https://www.ncbi.nlm.nih.gov/, last accessed May 25, 2017), and Ensembl Plants (http://plants.ensembl.org/Triticum_aestivum/Info/Index, last accessed May 25, 2017) websites, with *Arabidopsis* and rice NOX sequences as queries. We identified each NOX member by predicting the conserved domains. For further information, we analyzed some physicochemical parameters, predicted the subcellular localization and the numbers of transmembrane helix, and performed amino acid sequence alignment (see Supplementary Information S1 for detailed procedures).

### Chromosomal location and exon/intron structure analysis

Using Adobe_Photoshop_CS6 software, a total of 46 candidate genes were mapped to 18 different chromosomes according to the information from scaffolds and Gene ID reported in IWGSC and NCBI websites. The exon/intron logos of individual *NOX* and *FRO* genes were obtained from the Gene Structure Display Server (http://gsds.cbi.pku.edu.cn) by aligning the coding or cDNA sequences with their corresponding genomic DNA sequences.

### Sequence alignment and gene structure analysis

The phylogenetic tree of wheat NOX and FRO family members was constructed with MEGA 6.06. The logos of domain organization were obtained from EMBL-EBI (http://pfam.xfam.org/search#tabview=tab1) or SMART (http://smart.embl-heidelberg.de/) websites and were amended with Adobe_Photoshop_CS6. The four conserved domain motifs, namely NADPH_Ox, Ferric_reduct, FAD_binding_8, and NAD_binding_6 in each NOX sequence, were generated by MEME suite (http://meme-suite.org/) (see Supplementary Information S1 for detailed procedures).

### Phylogenetic relationships of NOX and FRO gene families in wheat and seven other plant species

Multiple sequence alignments and the phylogenetic relationship analysis of NOX and FRO gene families from eight plant species were performed using MEGA 6.06. Meanwhile, the non-synonymous (*K*a) and synonymous (*K*s) in paralogous and/or orthologous gene pairs from four species were also estimated using the bioinformatics software PAMLX 1.2 (see Supplementary Information S1 for detailed procedures).

### Subcellular localization analysis

The subcellular location of wheat NOXs was examined in rice protoplasts using transient transformation systems with some modifications (Zhang et al., 2011); at least three monoclones were sent for sequencing upon gene cloning and vector construction for each examined gene (see Supplementary Information S1 for detailed procedures).

### Prediction and functional analysis of cis-regulatory elements

We selected 2,000-bp genomic DNA sequences upstream of the transcriptional start sites of *TaNOXs* as the promoter sequences (named *TaNOX-pros*) to analyze the cis-acting elements using the databases: PlantCARE (http://bioinformatics.psb.ugent.be/webtools/plantcare/html/) (Lescot et al., 2002) and PLACE (http://www.dna.affrc.go.jp/PLACE/). Then, the activity of the promoters was analyzed by dual luciferase reporter assay (Zhang et al., 2011; Gu et al., 2013). Three or more monoclones were sent for sequencing to identify each promoter sequence and related vector construction. All values were shown as the average of the data collected from five or more replicates (see Supplementary Information S1 for detailed procedures).

### Plant materials, treatments, expression profiles, and coexpression network analysis

Tissue-specific expression profiles, inducible expression profiles of TaNOX genes, and coexpression analysis between TaNOXs and other genes in wheat (*Triticum aestivum* cv. Chinese Spring) were performed using the online Genevestigator v3 (https://genevestigator.com/gv/) and/or by qRT-PCR with *TaActin* (AB181991.1) and *TaGAPDH* (ABS59297.1) as the internal transcript level controls. The wheat seedlings were harvested from different developmental stages for tissue-specific expression profiles analysis. At the same time, the wheat seedlings treated with abiotic stresses and hormones were also collected for inducible expression profiles and coexpression network analysis. Total RNA was extracted from different samples using RNAiso TM Plus (Takara, Dalian, China) performance, and the subsequent quantitative real-time PCR (qRT-PCR) analysis was referenced to our previous study (Li et al., 2014). The results were presented as heat maps, histogram, and/or also as table lists. All the expression levels represent the mean ± SD of data collected from three independent experiments with each having three or four replicates (see Table S6 and Supplementary Information S1 for detailed procedures).

## Results

### Identification of NOX family genes in wheat genome

**Figure 1:**
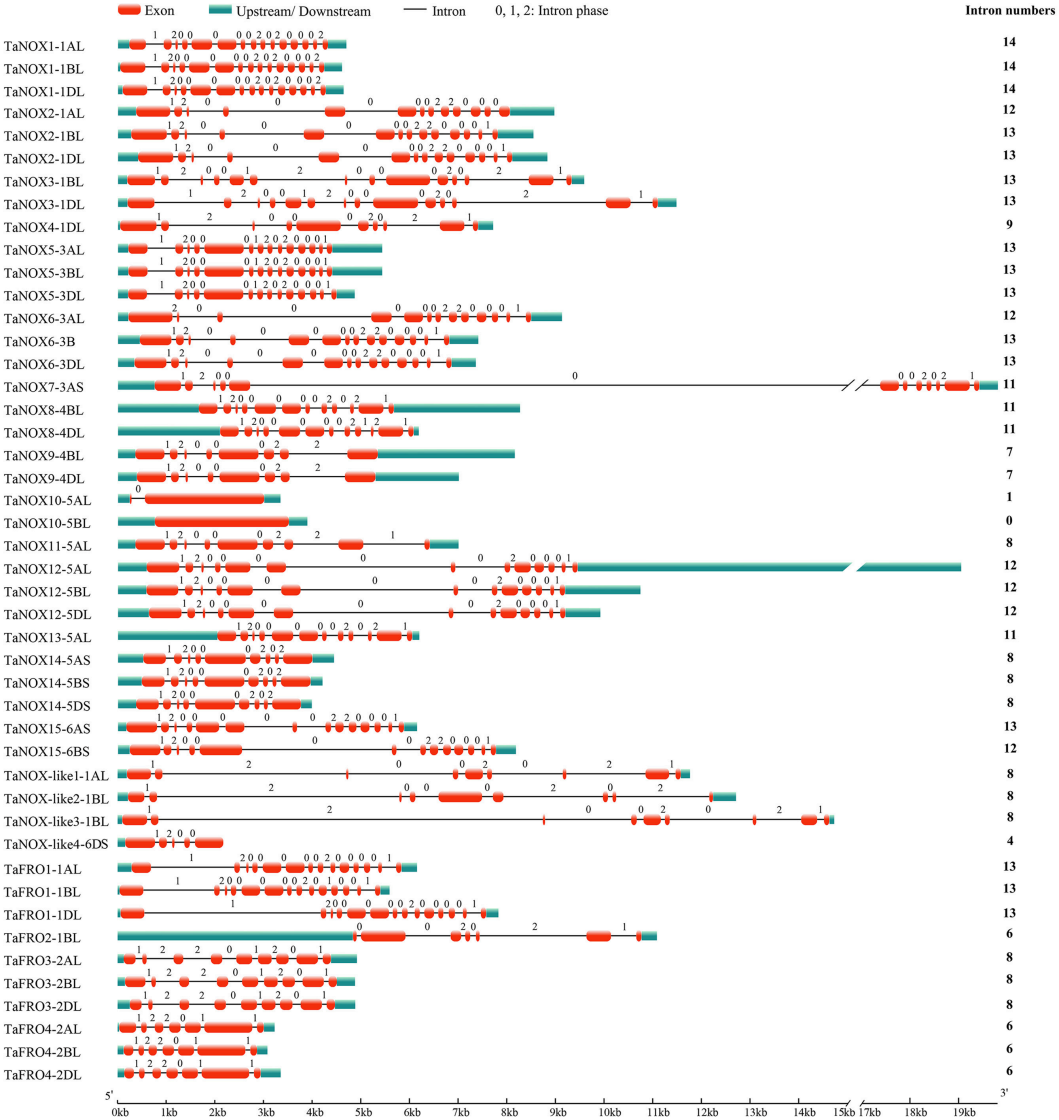
The exon/intron structures of NOX family genes in wheat. The numbers 0, 1, and 2 represent the phase of each intron in the sequence.

**Figure 2:**
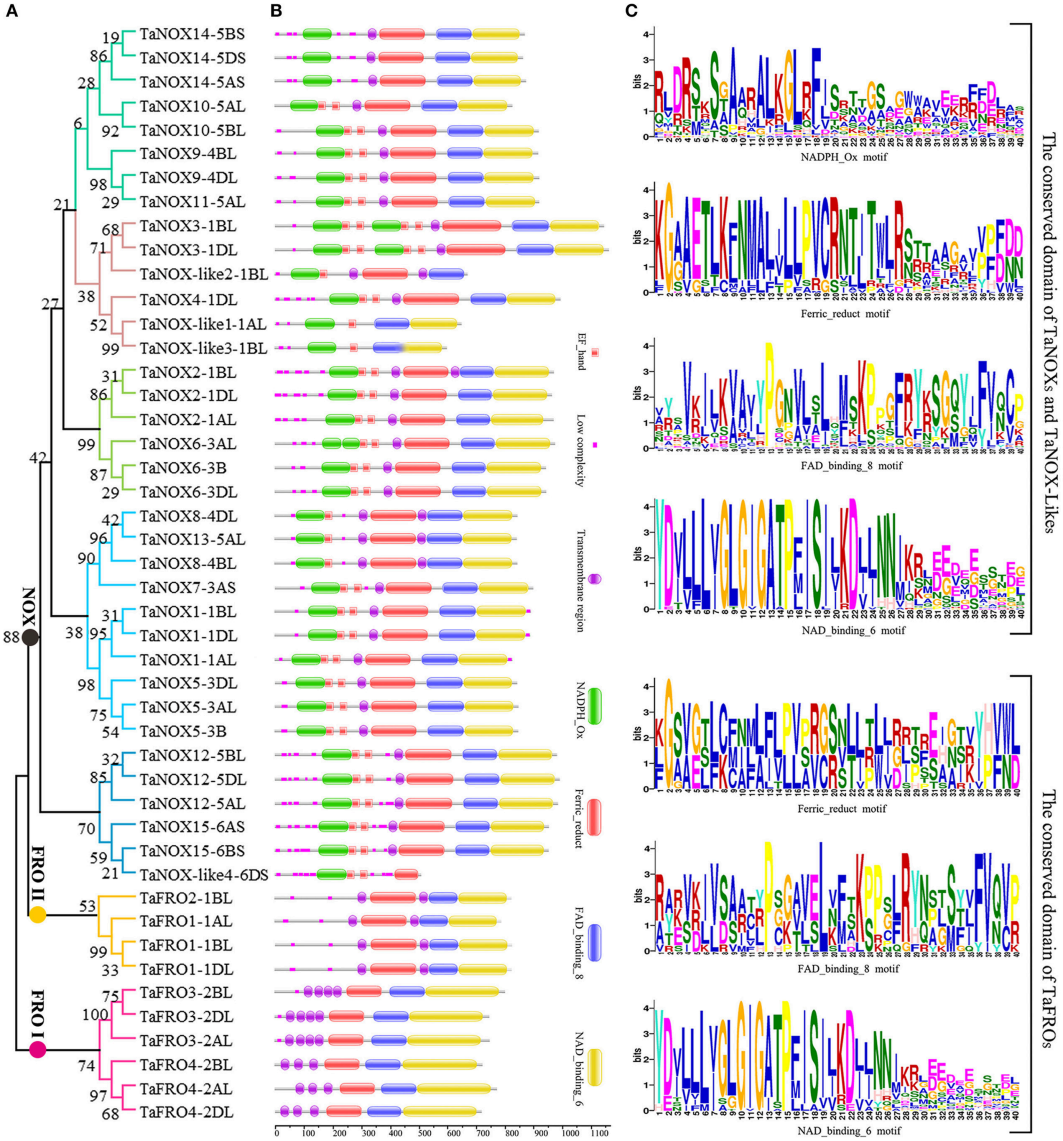
Phylogenetic relationship, domain organization, and conserved motifs analysis of NOX family proteins in wheat. **(A)** The unrooted maximum-likelihood phylogenetic tree of TaNOXs and TaFROs family members. Numbers above the nodes represent bootstrap values from 1,000 replications. **(B)** Domain organization of the TaNOXs and TaFROs. The logos of domain organization were obtained from EMBL-EBI and SMART websites and were amended with Adobe_Photoshop_CS6. **(C)** Four conserved motifs, namely NADPH_Ox, Ferric_reduct, FAD_binding_8, and NAD_binding_6, from all 46 NOX proteins in wheat. The logos of four conserved domain sequences were obtained from MEME Suite website. The bit score means information content of each position in the amino acid sequence.

A hidden markov model (HMM) search was performed to investigate and characterize the NOX gene family in wheat genome, and a total of 46 genes including 36 NOX and 10 FRO candidates were identified (Table S1). These NOX and FRO candidates can be divided into three types: TaNOXs, TaNOX-likes, and TaFROs based on their chromosome localization and domain composition (Table S1, Figures 1, 2). The homologous genes from different subgenomes (A, B, and D) were assigned the same number in gene denomination due to their similarity in gene structure and protein size. The typical NOXs (TaNOXs) have all four conserved domains, namely NADPH_Ox (Pfam accession number PF08414), Ferric_reduct (PF01794), FAD_binding_8 (PF08022), and NAD_binding_6 domain (PF08022). The TaNOX-likes have NADPH_Ox domain but lack one or two other domains, whereas the TaFROs lack NADPH_Ox domain but have the other three domains (Figure 2). Intriguingly, the NOX and FRO family genes in wheat genome are non randomly distributed across the chromosomes. It seems that FRO candidates are mainly distributed on Chr 2 and less on Chr 1; however, all the predicted NOX candidates are mainly distributed on Chr 1, followed by Chr 5, 3, 4, and 6, and none of the NOX members on Chr 2 (Figure S1). Moreover, the characteristics of gene structure and protein size are quite different between the NOX and FRO members (Figure 1, Table S1). TaNOXs are much bigger than TaFROs, whereas the TaNOX-likes are the smallest.

### Gene structure and domain composition of the wheat NOX family proteins

As can be seen in Figure 1, the gene structures are quite diverse between the *TaNOXs* and *TaFROs* with different intron numbers for different genes except for the certain homologous genes from different subgenomes (A, B, and D). On the whole, the gene constructions became more and more complicated from *TaFROs* to *TaNOXs* for increasing exons and introns. As a special type of NOX, *TaNOX-likes* are much simple with less exons and introns. According to the phylogenetic tree (Figure 2A), we found that all the homologous genes assigned as the same number in gene denomination were generally clustered into one group earlier than the others, and all the NOX family members could be classified into three big branches: FRO I (including TaFRO3 and 4) on the Chr 2, FRO II (including TaFRO1 and 2) on the Chr 1, and NOX (including TaNOXs and TaNOX-likes) on the Chr 1, 3, 4, 5, and 6, respectively.

As shown in Figure 2B, almost all the typical NOXs contain one or two transmembrane regions, one to four calcium-binding EF-hand motifs, and four conserved constructions, namely one or two typical NADPH_Ox domain, one Ferric_reduction domain, one FAD_binding_8 domain, and one NAD_binding_6 domain. Only TaNOX14 does not have an EF-hand motif (Figure 2B). All TaFROs possess the conserved domains that the typical TaNOXs have but lack the NADPH_Ox domain and the EF-hand motif. In contrast, the TaNOX-likes have only one or two of the three conserved domains similar to TaFROs possession, besides the typical NADPH_Ox and EF-hand motifs. Furthermore, the four functional domains exhibited to be all considerably conserved in construction, and the distribution of amino acid residues in every domain is quite similar but not identical among the NOX members (Figure 2C). Four conservative amino acid residues Y, G, G, and P presented with Y × × G × G × × P motif in the 5′end sequence of NAD_binding_6 domain from TaFROs were the same as those from the TaNOXs and TaNOX-likes. There were also other quite conserved residues, for example, the P in FAD_binding_8 domain and the G in Ferric_reduct domain, as indicated in capital letters in Figure 2C. However, the conservatism of amino acid residues in NADPH _Ox domain was lower than the other three conserved domains. Taken together, all the results mentioned above imply a closer evolutionary relationship between TaFROs and TaNOXs (Figure 2).

### Subcellular localization of the wheat NOX family proteins

**Figure 3:**
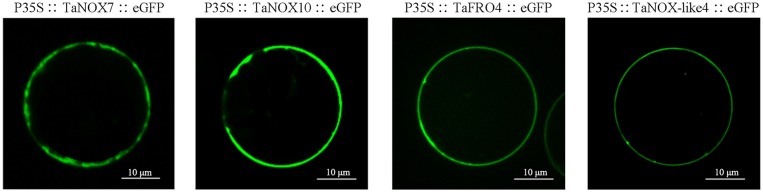
Subcellular localization of TaNOXs. Analysis of the subcellular location was performed using rice protoplast transient transformation systems. The constructs of TaNOX7::eGFP, TaNOX10::eGFP, TaFRO4::eGFP, and TaNOX-like4::eGFP were all under the control of the constitutive 2 × 35S promoters (cauliflower mosaic virus [CaMV]) in pTF486 vectors; at least three monoclones were sent for sequencing upon gene cloning and vector construction for each gene.

As shown in Table S1, all the NOXs including TaNOXs, TaNOX-likes, and TaFROs are located in the plasma membrane of cells as predicted by the Plant-mPLoc. Although no typical transmembrane helixes were identified in TaNOX-likes, these proteins were also predicted to be located at the plasma membrane. To verify the subcellular localization of TaNOXs, the full-length cDNA sequences of four genes, *TaNOX7-3AS, TaNOX10-5BL, TaFRO4-2BL*, and *TaNOX-like4*, were cloned and analyzed by a transient transformation system. As shown in Figure 3, the proteins encoded by the four genes are all located on the plasma membrane as expected.

### Systematic evolutionary relationship of NOX gene family in wheat

**Figure 4:**
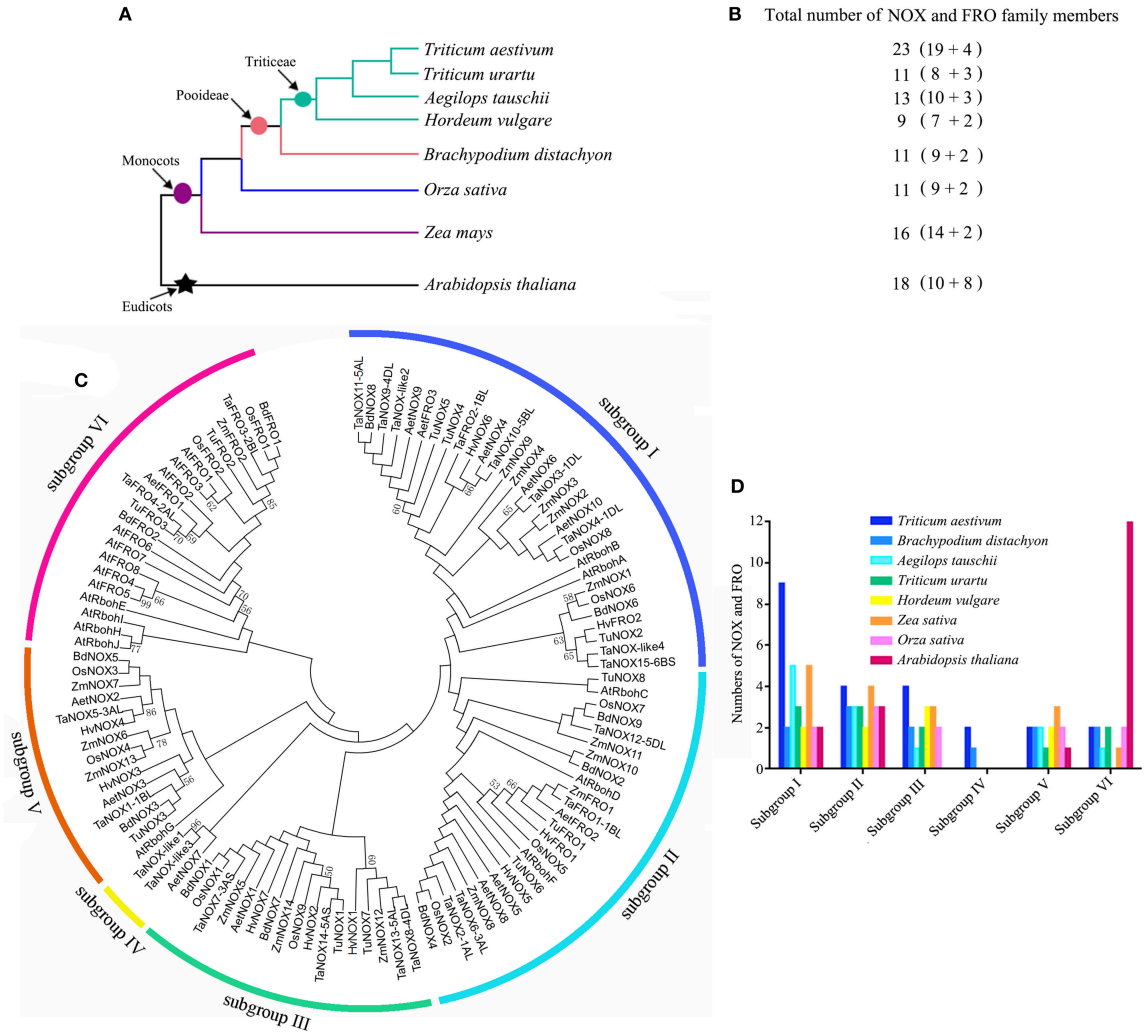
Systematic evolutionary relationships of NOX and FRO gene families in eight plant species. **(A)** Evolution relationship among the eight grass plant species. **(B)** The numbers of NOXs and FROs in each species. **(C)** Phylogenetic tree of NOX and FRO family members in the eight plants. **(D)** The number of NOXs in the subgroups I–VI.

To comprehensively dissect and characterize the evolutionary relationship of NOX gene family in wheat, a total of 112 NOX and FRO homologs were identified and selected from wheat and other seven plant species for the multiple sequence alignments and phylogenetic relationship analysis (Figure 4; Table S2). The evolutionary relationships between wheat (*T. aestivum*) and seven other species were presented in Figure 4A, and the numbers of NOX and FRO members were provided in Figure 4B. All the members of NOX and FRO families could be classified into six subgroups I–VI based on the difference of protein topological structure (Figure 4C). The wheat TaNOXs and TaFROs are distributed in every subgroup but dominantly distributed in subgroup I, whereas, the *Arabidopsis* AtNOXs (AtRbohs) and AtFROs are mainly distributed in subgroup VI (Figure 4D). The members within a certain subgroup exhibited a high identical percentage of amino acid sequences as shown in Table S3.

To estimate evolutionary relatedness and divergence time of NOX family genes between wheat (*T. aestivum*) and its diploid relatives: *Aegilops tauschii, Triticum urartu*, and *Hordeum vulgare*, the rates of non synonymous (*K*a) and synonymous (*K*s) nucleotide substitutions and their ratios (ω = *K*a/*K*s) of four putative paralogous and 19 orthologous gene pairs of NOXs were calculated using the bioinformatics software pamlX (Table S4). As can be seen, the majority of *K*a/*K*s ratios (ω) are more than 1 among the paralogous gene pairs, indicating that the NOX family genes have undergone strong positive selection pressure for functional expansion, and the duplication event was estimated to have occurred 3.16630–6.2533 Mya. On the contrary, the values of ω are almost less than 1 among the orthologous gene pairs, suggesting that the NOX family genes have undergone strong negative selection pressure for functional conservation, and the duplication event was estimated to have occurred 0.0060–0.6466 Mya.

### Development and tissue-specific expression of NOX family genes in wheat

**Figure 5:**
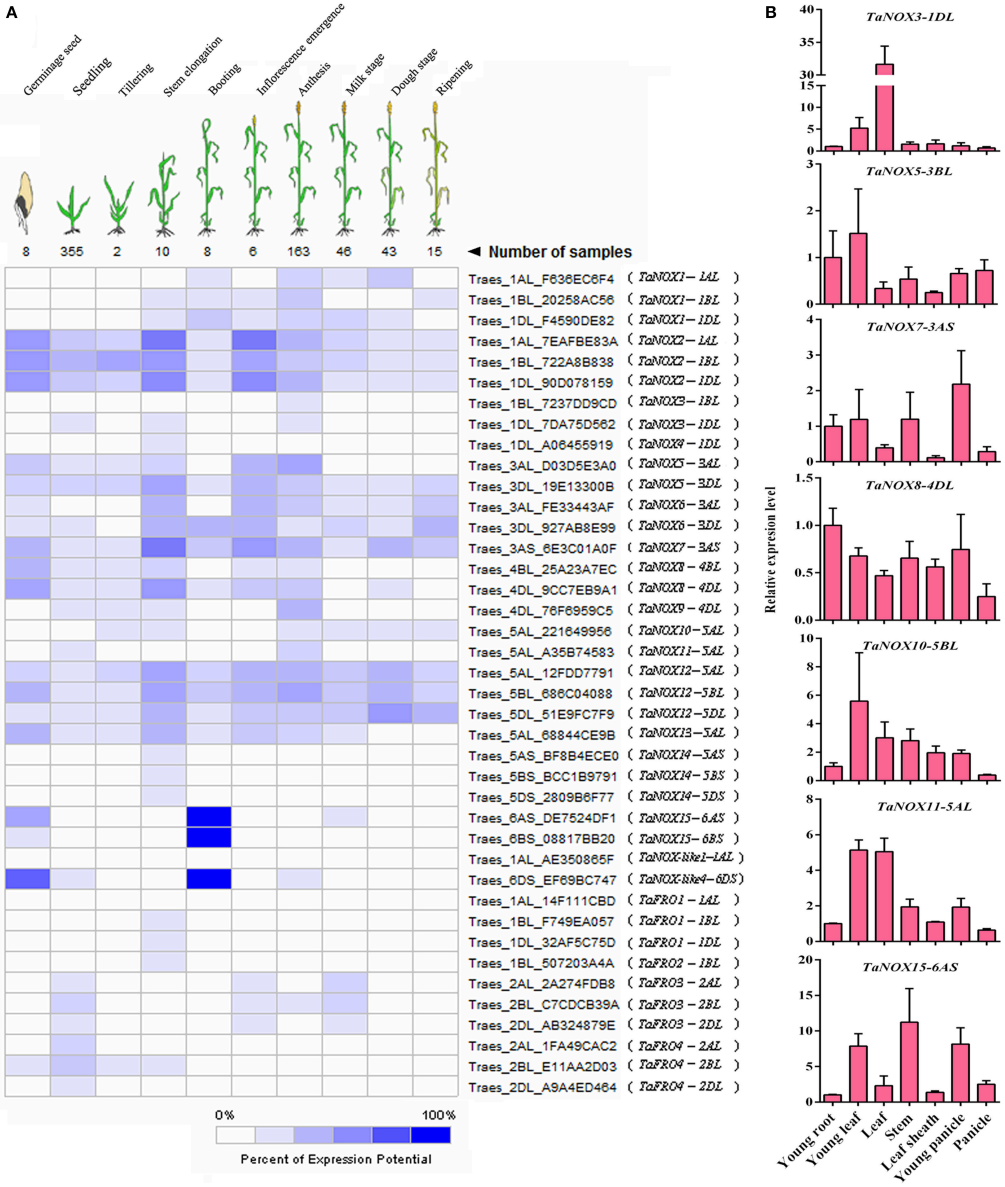
Expression patterns of NOX and FRO family genes in wheat at different developmental stages and in different tissues. **(A)** The developmental expression pattern analysis of NOX and FRO family genes in 10 developmental stages during wheat growth. All the data were selected from the Ta_mRNASeq_WHEAT_GL-0 database. **(B)** Expression profiles of seven wheat NOX genes in seven different tissues. The seven tissues are as follows: the young leaf and young root came from 2-week-old seedlings; the leaf, stem, leaf sheath, and young panicle came from the booting stage; the panicle came from the inforescence emergence stage. The developmental expression pattern for each NOX or FRO family gene was performed by the three independent experiments with three replicates for each experiment.

To clarify the expression profiles of NOX family genes during the development of wheat, a set of microarray data for the gene expression were obtained from Genevestigator v3. To simplify the phraseology in the following experiments, the homologous genes located on different subchromosomes (Chr A, B, and C) are referred to as *TaNOXx*; for example, *TaNOX1-1AL, -1BL*, and/or *-1DL* were all named as *TaNOX1*. The expression levels of *TaNOXs* at 10 developmental stages were presented in Figure 5A. As can be seen, different *TaNOXs* exhibited different expression patterns with some genes dominantly expressed at certain stages. For example, the genes *TaNOX2, 7*, and *12* are widely expressed in whole developmental stages with the highest in stem elongation and inflorescence emergence stages, whereas *TaNOX15* and *TaNOX-like4* are mainly expressed in germinating seeds and at booting stage. The expression of *TaFROs* has more specificity in wheat. For instance, *TaFRO1* and *TaFRO2* are only expressed at stem elongation stage, whereas *TaFRO3* is mainly expressed at young seedling, inflorescence emergence, and milk stages (Figure 5A).

The tissue-specific expression profiles of *TaNOXs* were examined by qRT-PCR (Figure 5B). Seven wheat tissues from different developmental stages were used for the analysis. According to the tissue-expression profiles obtained from Genevestigator v3, seven TaNOX genes, including *TaNOX3, 5, 7, 8, 10, 11*, and *15*, were selected as the candidates to study the tissue specificities of the genes in wheat. The results showed that every gene had its obvious tissue specificities in expression. The expression of *TaNOX3* is the highest in leaves at booting stage, whereas the expression of *TaNOX5, 10*, and *11* is the highest in young leaves at seedling stage, *TaNOX7* in young panicle at booting stage, *TaNOX8* in young roots at seedling stage, and *TaNOX15* in stems at booting stage. All these results indicated that the expression of each *NOX* gene is unique and displays a strong spatio-temporal and tissue specificity in wheat.

### Analysis of the promoters of NOX genes and their response to exogenous treatments

**Figure 6:**
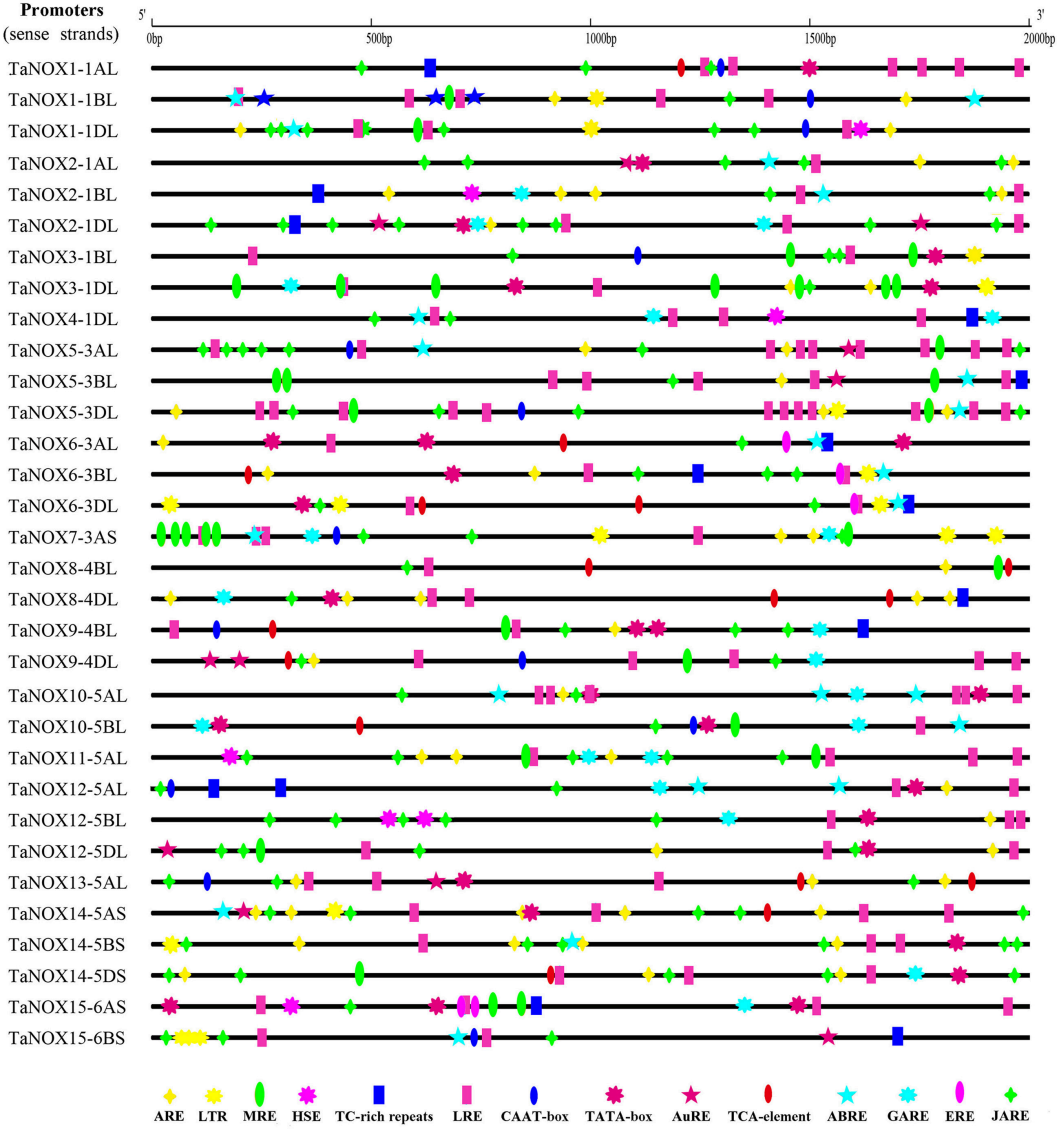
The cis-elements responding to abiotic/biotic stresses and hormone treatments in the sense strands of *TaNOX* promoters. The cis-elements are as follows. Anaerobic responsive element (ARE): ARE and GC motif; LTR: low-temperature responsiveness; MYB-responsive element (MRE): MBS and MRE; HSE: heat stress responsiveness; TC-rich repeats: defence and stress responsiveness; light-responsive element (LRE): G-box, SPI, MNF1, 4cl-CMA2b, GT1-motif, ACE, and BoxI elements; CAAT-box: common cis-acting element in promoter and enhancer regions; TATA-box: core promoter element around−30 bp of transcription start; auxin-responsive element (AuRE): AuXRR core and TGA element; TCA element: salicylic acid (SA) responsiveness; abscisic acid-responsive element (ABRE): ABRE, motif IIb and CE3 elements; gibberellins (GA)-responsive element (GARE): GARE, TATC-box and p-box; ERE: ethylene-responsive element; methl jasmonic acid (MeJA)-responsive element (JARE): TGACG motif and CGTCA motif.

The promoters of all *TaNOX* genes were analyzed, and the cis-acting elements existing in the promoters were predicted by the databases of PlantCARE, PLACE, and Neural Network Promoter Prediction. In total, 31 cis-regulatory elements with the prediction score being greater than or equal to 5 remained and were depicted on the corresponding sequences. The elements responsive to environmental stresses and hormones were considered for further analysis (Figure 6, Figure S2). As can be seen, almost all the stress or hormone responsive elements are distributed randomly on the promoter sequences of the *TaNOX* genes. However, some cis-elements are distributed in clusters in certain promoters, for example, MBS-motif, TGACG-motif, and G-box in the promoters of *TaNOX7AS, TaNOX5-3AL* and *TaNOX5-3BL* genes, respectively, implying the essential roles of these elements in the expression of these genes. Interestingly, the numbers and distribution patterns of the cis-elements are also greatly varied among the promoters of homologous genes with the same number in gene denomination although their encoding proteins have an extreme similarity in amino acids sequence and domain construction (Figure 6). This means that the homologous genes might have different regulatory mechanisms in expression, implying their functional divergence during the polyploidization of wheat genome.

**Figure 7:**
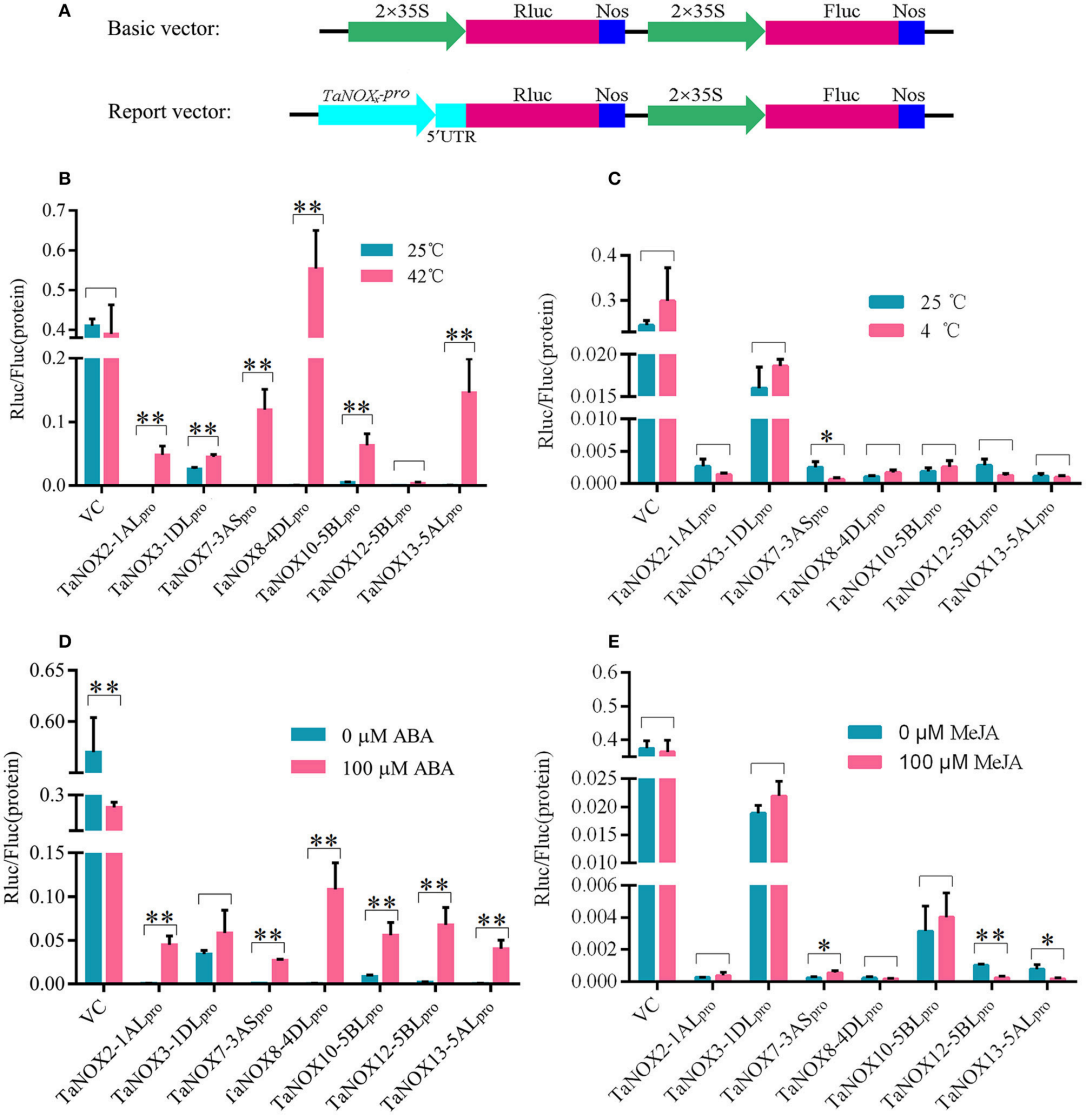
Activity analysis of the promoters of *TaNOXs* under temperature stress and hormone treatments. **(A)** Schematic representation of the basic and report vector constructs. **(B–E)** The relative activity of the promoters of *TaNOXs* under temperature stress and hormone treatments. The activity analysis of the promoters represents the mean ± SD of data collected at least seven replicates. *P < 0.05; **P < 0.01.

To get further insight into the regulation of gene expression of TaNOXs, the promoters of seven genes were cloned, and their activities in response to heat, cold, ABA, and MeJA treatments were determined using a dual luciferase reporter system. As shown in Figure 7, each promoter examined exhibited different levels of biological activities in response to different treatments. The activities of almost all the promoters of seven TaNOX genes could be greatly stimulated by heat (Figure 7B), whereas no marked changes in the activities were observed under cold stress compared with the controls. Only the activity of *TaNOX7-3AS* promoter was obviously decreased by cold (Figure 7C). ABA treatment also significantly stimulated the activities of the promoters of the TaNOX genes except for the promoter of *TaNOX3-1DL* (Figure 7D). Although the MeJA-responsive cis-elements are widely distributed in the promoters of the seven genes examined here, only the promoters of *TaNOX7-3AS, TaNOX12-5BL*, and *TaNOX13-5AL* responded to the exogenous MeJA treatment (Figure 7E). These results indicated that the responses of the TaNOX genes to environmental factors are quite different, and a complicated mechanism might be involved in the control of gene expression in wheat.

### Inducible expression profiles range of *TaNOXs* under a wide range of environmental stresses and hormone treatments

**Figure 8:**
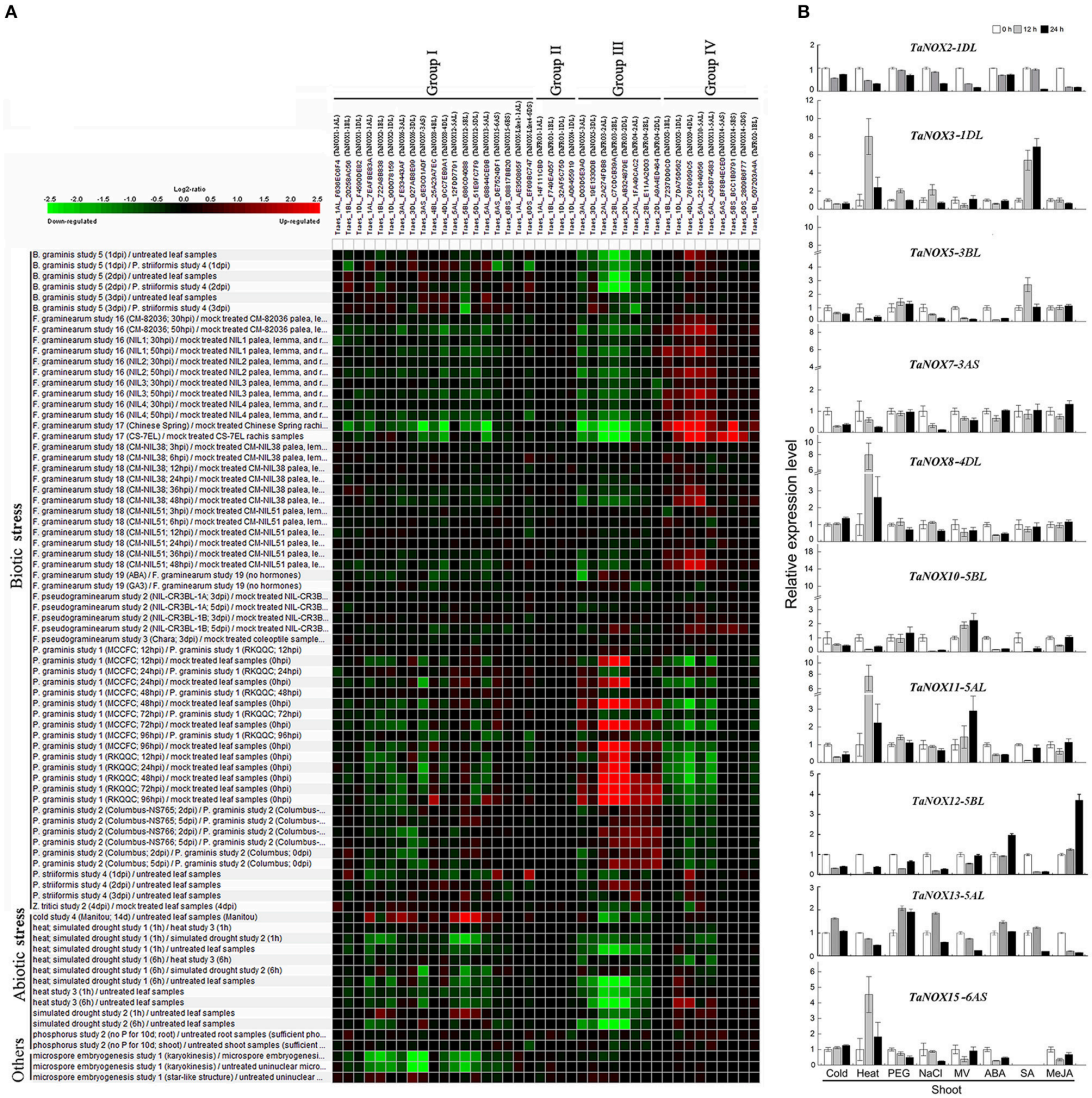
Inducible expression patterns of wheat NOX family genes under different biotic and abiotic stresses. **(A)** The inducible expression patterns of *TaNOXs* obtained from the database of Ta_mRNASeq_WHEAT_GL-0 as reported by Genevestigator v3. Results were given as heat maps in green/red coding that reflect relative signal values, where greener represents stronger down regulated expression and redder represents stronger up-regulated expression. **(B)** The inducible expression patterns performed by qRT-PCR under cold (4°C), heat (40°C), 20% PEG6000, salt (200 mM NaCl), and oxidative (30 μM MV) stresses and by ABA (100 μM), SA (500 μM), MeJA (100 μM) hormone treatments. Two-week-old seedlings were used for the analysis. The expression level of every NOX gene is the mean of results from three independent experiments, each having three or four replicates.

To further study the expression characteristics of wheat NOX family genes under suboptimal conditions, we carried out a comprehensive analysis using both the wheat microarray data in Genevestigator v3 and qRT-PCR experiment (Figure 8, Figure S3). As can be seen in Figure 8A, many NOX genes in wheat can respond to a number of environmental stresses. Different inducible expression patterns could be seen in different NOX genes responding to different biotic stresses such as *B. graminis, F. graminearum, P. graminis, P. striliformis*, and *X. translucens* and to different abiotic stresses such as cold, heat, drought, and salt. According to the expression patterns, the TaNOX family genes can be simply classified into four groups: Group I including *TaNOX1, 2, 6, 7, 8, 12, 13*, and *15* and *TaNOX-like1* and *4* was upregulated by cold but downregulated by almost all the other environmental treatments; Group II including *TaFRO1* and *TaNOX4* had no obvious responses to all the environmental treatments; Group III including *TaNOX5, TaFRO3*, and *4* was upregulated by *P. Graminis* attack but downregulated by all abiotic treatments and *B. graminis* and *F. graminearum* attacks; and Group IV including *TaNOX3, 9, 10, 11, 14*, and *TaFRO2* was upregulated by *B. graminis* and *F. graminearum* attacks but downregulated by *P. graminis* attack (Figure 8A).

The different responsive profiles of the NOX gene members to different abiotic stresses were also observed by qRT-PCR experiment (Figure 8B, Figure S3B). Here, only a more than two-fold difference in transcript levels was considered to be a true difference for the genes under treatments. Under cold (4°C) stress, most TaNOX genes were downregulated in both shoots and roots at 12 and 24 h time points of the treatment, whereas *TaNOX7* was upregulated in roots at 12 h time point. Similarly, most of the genes were downregulated by salt stress (200 mM NaCl) in both shoots and roots at 12 h and/or 24 h time point, whereas *TaNOX5* was upregulated in roots at 12 h time point. However, heat stress (40°C) markedly upregulated the transcript levels of *TaNOX3, 8, 11*, and *15* genes but downregulated *TaNOX2, 5, 7, 10*, and *12* genes in shoots. In contrast, except for *TaNOX12* and *13*, almost all the other genes examined were downregulated by heat in roots (Figure S3B). All the examined TaNOXs could be upregulated by the oxidative stress (30 μM MV) in roots. On the contrary, almost all TaNOX genes had no obvious changes by osmotic stress (20% PEG6000) in both roots and shoots except *TaNOX13* was upregulated in shoots. Furthermore, except *TaNOX7*, which showed no changes, and *TaNOX12*, which was upregulated, all the other genes were downregulated by ABA treatment (100 μM) in shoots, whereas except *TaNOX5*, which was greatly upregulated, and *TaNOX12* and *13*, which were downregulated, all the others showed no obvious changes in roots. With the treatment of MeJA (100 μM), the majority of *TaNOXs* had no significant changes, but *TaNOX12* in shoots and *TaNOX5, 7*, and *10* in roots were exhibited upregulation. For SA treatment (500 μM), the genes of *TaNOXs* exhibited more complicated expression profiles, and no distinct consistency in expression patterns was observed. Simply, it can be seen that *TaNOX3* and *5* genes were upregulated by SA in shoot, whereas *TaNOX8* and *10* were upregulated in roots. Although not all the NOX family genes were examined, the results obtained here suggested that each NOX member in wheat has its unique inducible expression profiles and thus has its own functional specificity in the plant stress responses.

### Coexpression relationships between the genes of *TaNOXs* and others

To further explore the biological functions of TaNOX genes, we generated the coexpression patterns of *TaNOXs* with others using the wheat microarray data in the database of Ta_mRNASeq_WHEAT_GL-0 from Genevestigator v3 (Figures S4–S6). Eight genes were selected for the analysis according to their inducible expression profiles. Interestingly, several genes showed obvious coexpression relationships with certain NOX genes either at 10 different developmental stages (Figure S4) or in 22 different tissues (Figure S5). These coexpressed genes are mainly involved in the substance transport, cell wall synthesis, redox reaction, phosphorylation, and/or gene expression regulation (Table S5). For instance, *TaNOX2* and *3* showed positive correlation with the genes encoding two S-locus lectin protein kinase family proteins; *TaNOX3, 7*, and *TaFRO3* are coexpressed with the genes encoding four leucine-rich repeat protein kinase family proteins. The eight NOX genes also exhibited distinct coexpression with other genes under a lot of biotic and abiotic stress conditions (Figure S6). These coexpression genes are widely involved in stress response, signal transduction, cell wall formation, protein degradation, substance transport, and transcriptional regulation (Table S5). For example, *TaNOX2* is highly coexpressed with a gene encoding *S*-adenosyl-l-methionine-dependent methyltransferase and a gene encoding ubiquitin-specific protease family C19-related protein, whereas *TaNOX3* is highly coexpressed with the genes encoding two brassinosteroid-Insensitive 1 precursor kinases and a gene encoding glutathione *S*-transferase tau 7.

**Figure 9:**
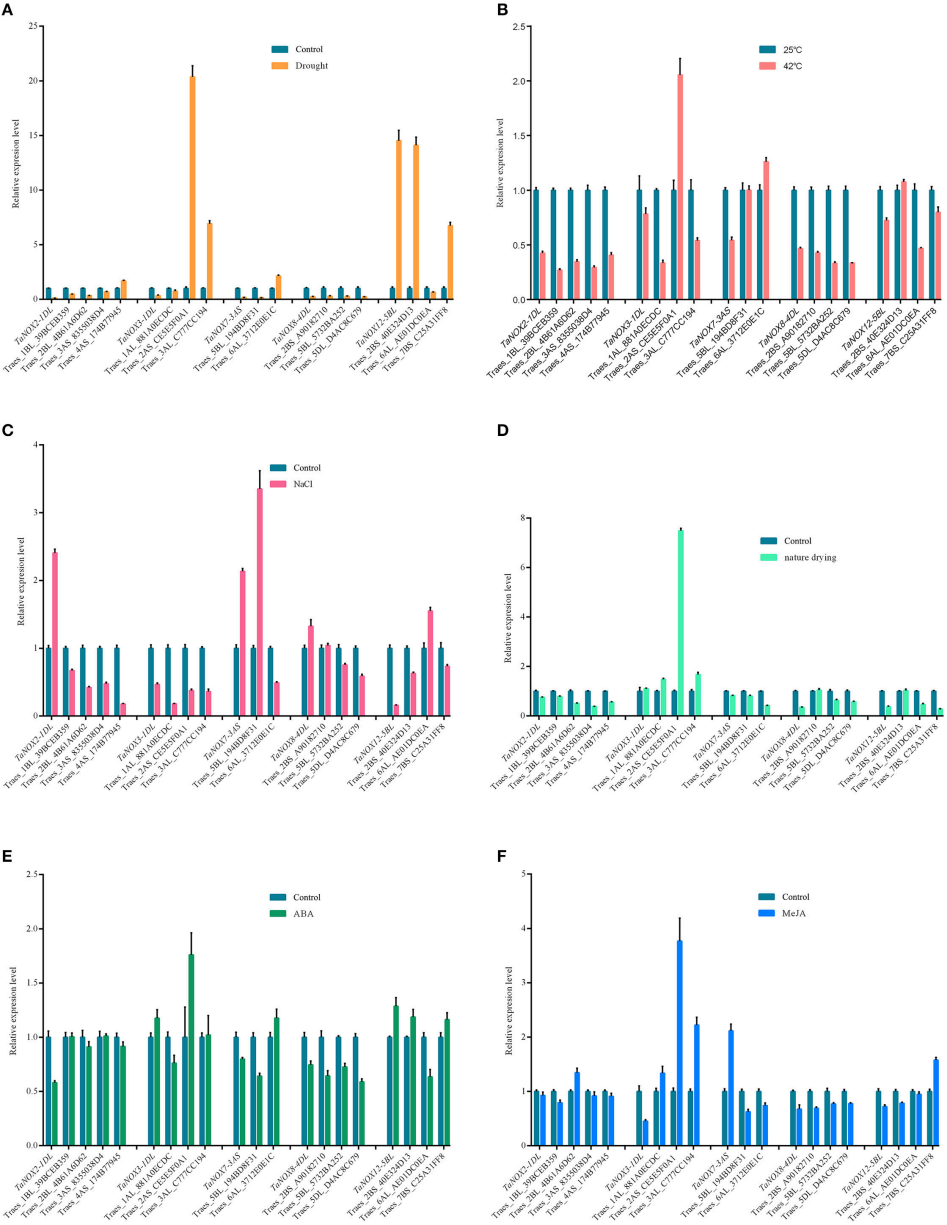
Expression profile analysis of coexpression genes with *TaNOXs* by qRT-PCR under different abiotic stress treatments on the shoot of wheat seedlings. The 7-day-old wheat seedlings treated with soil drought for 2 days **(A)**, 42°C for 24 h **(B)**, 200 mM NaCl for 6 h **(C)**, nature drying for 30 min **(D)**, 10 μM ABA for 6 h **(E)**, and 10 μM/L MeJA for 6 h **(F)**, respectively, were used as the materials for RNA extraction. Data are mean values from at least five replicates. Abbreviations and locus names of the coexpression genes used here are available in Table S5.

To better understand the coexpression relationships of the *TaNOXs* with other genes under abiotic stresses, we chose some genes as targets for qRT-PCR. According to the results of tissue and inducible expression profiles, five TaNOX genes and six stress conditions were selected for the experiment. Here, more than two fold difference in transcript levels was considered to be a true difference for the genes as well. As shown in Figure 9, most of the genes selected exhibited significant coexpression patterns with the *TaNOXs* under a certain stress condition. Only a few genes had an opposite response with the *TaNOX* gene under some special stress. As can be seen, *TaNOX2* exhibited an opposite response with its predicted coexpression genes only under salt stress. For *TaNOX3*, except under drought and MeJA treatments, it seems that only one predicted gene was not obviously coexpressed with it under four other stress conditions. The distinct coexpression relationships between *TaNOX7* and the predicted genes could be observed under heat, natural drying, and ABA treatments. By contrast, *TaNOX8* exhibited positive coexpression with the predicted genes under all the six stress treatments. Except drought and salt stresses, *TaNOX12* also showed obvious coexpression with its predicted genes examined under other four treatments. These results indicated that the coexpression relationships of *TaNOXs* with the predicted genes might be stress dependent, implying the specific but complicated functions of NOX genes in wheat development and/or stress responses.

## Discussion

### Wheat NOXs are diverse in members and structures with a complicated evolution history

As is well-known, common wheat (*T. aestivum*) is a hexaploid species with three closely related subgenomes (A, B, and D). In this study, a total of 46 NOX family genes, which encode 15 TaNOXs, 4 TaNOX-likes, and 4 TaFROs, were identified according to the sequence analysis and domain composition (Table S1). Phylogenetic analysis showed that the homologous genes from different subgenomes for a certain NOX protein always clustered together first, as expected (Figure 2). Interestingly, not every protein has three homologous genes distributed on the subchromosomes A, B, and D. For example, TaNOX3 includes two homologous genes (*TaNOX3-1BL* and *-1DL*) but *TaNOX4* has only one (*TaNOX4-1DL*) (Figure 1). This means that their homologs on a certain subchromosome might be lost during the long-term evolution and natural selection. In fact, many previous studies have shown that in an allopolyploid species, the phenomenon of genovariation including gene rearrangement, structural variation, DNA sequence loss or amplification, and transposon activation occurred frequently during the polyploidization of genome (Jackson and Chen, 2010).

As suggested earlier, gene duplication, fusion, and/or exon shuffling are commonly occurred in eukaryotes for the biological diversity and functional divergence of certain gene family during evolution in plants (Morgante et al., 2005; Van de Peer et al., 2009; Kaessmann, 2010; Magadum et al., 2013). This is also involved in the expansion of NOX gene family in plants (Chang et al., 2016). The typical NOXs of wheat exhibited high similarity in the sequences of the conserved domains but obvious diversity in gene structure and protein size (Table S1, Figures 1, 2). TaFROs also showed very high similarity in sequences of the conserved domains compared with those of TaNOXs although there are still some differences in same conserved amino acids (Figure 2), implying an evolutionary relationship between TaFROs and TaNOXs. In fact, it was suggested that FRO proteins are the ancestral forms of NOXs in both animals and plants (Chang et al., 2016). Interestingly, although the structures of TaNOXs and TaFROs are conserved, they have undergone a diverse structural evolution. It can be seen that the numbers and positions of intron and exon of the genes became more and more complicated from TaFROs to TaNOXs (Figure 1), exhibiting a structural diversity of NOX family genes in wheat, and the evolutionary mechanisms such as gene duplication, gene fusion, and exon shuffling might be also involved in the evolution of wheat NOX gene family. Moreover, although TaFROs are the ancient forms of TaNOXs, the *TaFROs* genes located on Chr 1 and Chr 2 seem to be somewhat different in evolution and function. The *TaFROs* located on Chr 1 (*TaFRO1* and *TaFRO2*) and Chr 2 (*TaFRO3* and *TaFRO4*) could not be clustered into the same subgroup within the phylogenetic tree. The *TaFROs* located on Chr 1 were clustered into the small subgroup and were closer to the *TaNOXs* and *TaNOX-likes* than the *TaFROs* located on Chr 2 (Figure 2A), indicating their differential but complicated evolution of the two types of TaFROs. The TaFROs encoded by the genes located on Chr 2 exhibited different numbers and arrangement of transmembrane regions and different sizes of the three conserved domains compared with those located on Chr 1 (Figure 2), showing their diversity in protein structure and probably also in function. Moreover, it seems that the arrangement and similarity of Ferric_reduct domains and transmembrane regions of the TaFROs encoded by the genes located on Chr 1 are more similar to those of the typical TaNOXs, further suggesting that the TaFROs encoded by the genes located on Chr 1 perhaps are closer to TaNOXs in genetics than those on Chr 2 and the TaFROs on Chr 2 might be the more ancient forms of TaNOXs in wheat.

As reported earlier, typical NOXs only exist in terrestrial plants evolved from the ancient FROs by gaining a NADPH_Ox domain (Chang et al., 2016). The NADPH_Ox domain endowed NOX with a new function of producing ROS as a defence mechanism. Although the FROs in lower organisms are responsible for both ROS production and iron uptake (Aguirre et al., 2005), a number of increasing literatures have shown that in higher plants, NOX-mediated ROS production is mainly responsible for the stress tolerance and development regulation (Kaya et al., 2014; Gupta et al., 2017; Yamauchi et al., 2017), whereas FROs are mainly responsible for iron uptake and iron homeostasis (Sperotto et al., 2010). Furthermore, TaNOXs perhaps will become more complicated in construction; for example, TaNOX3 possessing two NADPH_Ox domains, and this is different from the others that just possesses one NADPH_Ox domain in animo acid sequences (Figure 2B). Further study is needed to check whether it has some biological significance.

The four TaNOX-likes are very special because they have the NADPH_Ox domain but lack one or two other conserved domains that the typical TaNOXs have (Figure 2), and although no typical transmembrane helixes were identified in these proteins, they are also located in the plasma membrane (Figure 3). The analysis for phylogenetic relationships showed that TaNOX-like1 and 3 are much closer to TaNOX4, whereas TaNOX-like2 is much closer to TaNOX3 and TaNOX-like4 is much closer to TaNOX15 (Figures 3, 4), indicating that they might derive from these three typical TaNOXs, respectively, probably by DNA sequence loss during evolution. As no NOX-like proteins were identified in other plant species and no experiments have been done on them, their biological functions are unclear. All this information suggest that wheat NOXs underwent a complicated evolutionary history during the gene expansion and functional divergence.

### Wheat NOXs exhibit a great specificity in expression and play vital roles in both the plant growth regulation and stress response

Specific expression is a common characteristic of the genes of a certain protein family in plants, which often reflects the cross-talk and/or difference in functions of the family members. Many studies have shown that different NOXs have different functions both in animals and in plants (Bedard and Krause, 2007; Kaye et al., 2011). In *Arabidopsis*, 10 NOXs were found, and these NOXs participate in different biological processes and/or stress responses with obvious tissue specificity in the gene expression (Maruta et al., 2011; Chaouch et al., 2012; Kaya et al., 2014; Xie et al., 2014). However, in wheat, although it was reported that NOXs could ameliorate the nickel-induced oxidative stress (Hao et al., 2014), to date, only two genes have been reported functioning in brown rust infection (Dmochowska-Boguta et al., 2013). In the present study, both the results obtained from database research and qRT-PCR analysis indicated that TaNOXs widely function in the plant growth regulation and stress response (Figures 5–8, Table 1). For example, *TaNOX2* is expressed in a whole plant with dominantly existing in stem elongation and inflorescence emergence stages, and its transcripts were greatly affected by a set of biotic and abiotic stresses, implying its vital roles in plant growth regulation and also stress responses. TaNOX2 is more similar to *Arabidopsis* AtRbohD and AtRbohF and rice OsNOX2 (OsRbohA) in phylogenetics (Figure 4). AtRbohD and AtRbohF are all expressed throughout the plant (Sagi and Fluhr, 2006) and are essential not only for the hypersensitive response but also for the ABA-, JA-, and ethylene-induced signaling pathways (Suhita et al., 2004; Desikan et al., 2006; Zhang et al., 2009; Maruta et al., 2011), disease resistance (Chaouch et al., 2012), salt stress tolerance (Xie et al., 2011), and endosperm development (Penfield et al., 2006). *OsNOX2* was also found to be expressed in the whole plant and plays crucial roles in both growth regulation and drought stress tolerance (Wang et al., 2016). TaNOX8 and TaNOX12 are also much close to AtRbohD, AtRbohF, and OsNOX2 (Figure 4), and as expected, the two wheat genes exhibit very similar expression profiles with *TaNOX2*, although they are somewhat different under a certain stress condition, implying their functional identity or coordination. In contrast, *TaNOX3* showed marked tissue and/or development specific expression (Figure 5, Table 1) with a great response for many different treatments (Figures 7B, **8**, Table 1), suggesting somewhat specificity in functions. TaNOX3 is much closer to *Arabidopsis* AtRbohB, which was found to be specifically expressed in root and elongation zone and to be involved in seed after ripening (Sagi and Fluhr, 2006; Muller et al., 2009). Similar to TaNOX3, the TaNOXs including TaNOX4, 9, 10, and 11 are also much closer to AtRbohB in phylogenetics, but these TaNOXs exhibit varied tissue and/or development expression profiles as well as the inducible expression (Figures 5, 7, 8, Table 1), implying somewhat divergence in functions among these NOX proteins. *TaNOX15* also shows notable tissue- and development-specific expression with more transcripts in stems at booting stage (Figure 5, Table 1), but the transcripts were less induced by different treatments (Figure 8, Table 1). TaNOX15 has high identity in amino acid sequence with Arabdopsis AtRbohC, which was reported participating in root-hair-tip growth and specifically expressed in roots (Sagi and Fluhr, 2006; Takeda et al., 2008), which means that it has different functions in wheat as AtRbohC has in *Arabidopsis*.

**Table 1:** Expression specificity and functional diversity of TaNOXs in wheat.

**TaNOXs**	**Tissue specificty**	**Expression level**	**Functions and sensibility**
TaNOX1	Booting; anthesis; dough stage	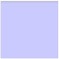	Sensitive to biotic and abiotic stress; cold ↑
TaNOX2	Seed germination; stem longation; inflorescence emergence	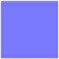	sensitive to biotic and abiotic stress and ABA, SA, MeJA treatment; NaCl ↑, cold ↑, drought ↑, heat ↑
TaNOX3	Seedling; stem longation; anthesis	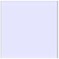	Sensitive to biotic and abiotic stress and SA treatment; drought ↑, cold ↓
TaNOX4	Stem longation	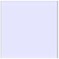	(Development regulation)
TaNOX5	Inflorescence emergence; anthesis	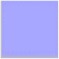	Sensitive to biotic and abiotic stress and ABA treatment
TaNOX6	Stem longation; inflorescence emergence	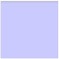	Sensitive to biotic and abiotic stress; (development regulation); cold↑
TaNOX7	Seed germination; Stem longation; inflorescence emergence	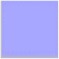	Sensitive to biotic and abiotic stress; ABA and MeJA treatment;
			(development regulation); drought↑, NaCl↑
TaNOX8	Seed germination; stem longation	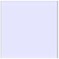	Sensitive to biotic and abiotic stress; heat↑
TaNOX9	Athesis	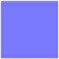	Sensitive to biotic and abiotic stress; heat ↑
TaNOX10	Whole plant (except seedling, seed, booting, and florescence emergence)	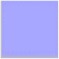	Sensitive to biotic stress and MeJA treatment
TaNOX11	Seedling; anthesis	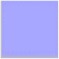	Sensitive to biotic stress
TaNOX12	Stem longation; athesis; dough stage	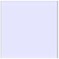	Sensitive to biotic and abiotic stress, ABA treatment; drought↑, cold↑, NaCl↓
TaNOX13	Seed germination; stem longation; inflorescence emergence	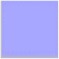	Sensitive to biotic and abiotic stress, ABA treatment; drought↑, cold↑
TaNOX14	Stem longation	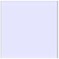	Sensitive to biotic stress (development regulation)
TaNOX15	Germinating seed; booting	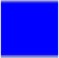	(Development regulation)

↑: upregulated; ↓: downregulated. 
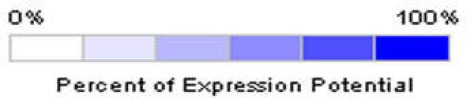

However, TaNOX7, 8, 13, and 14 exhibit much closeness to rice OsNOX1 (OsRbohB) and OsNOX9 (OsRbohH) (Figure 4), and all of them are sensitive to biotic stresses and ABA treatments (Figures 7B, 8, Table 1), implying their possible functions in plant immunity. It was reported that OsNOX1 (OsRbohB) involves in immune response (Nagano et al., 2016), whereas OsNOX9 (OsRbohH) regulates the ethylene-induced aerenchyma formation in roots (Yamauchi et al., 2017). Furthermore, TaNOX1 and TaNOX5 are much closer to OsNOX3, which was also found to play crucial roles in plant immune response (Yoshie et al., 2005; Nagano et al., 2016). TaNOX1 and TaNOX5 having similar functions require further study. Interestingly, due to the inducible expression profiles under a set of biotic and abiotic stresses, all the TaNOXs could be divided into four subgroups (Figure 8), implying their functional divergence between the groups but coordination within the same group. However, as discussed above, different TaNOXs exhibit strong spatio-temporal and tissue-specific expressions, demonstrating an obvious cross-talk and/or divergence in both biological roles and evolutionary relationship of the NOX family genes in wheat.

### Coexpression analysis reveals the wide functions but complicated regulatory mechanisms of the TaNOXs in wheat

Acting as the key producer of ROS, NOXs play vital roles in plant growth and development including root and root hair elongation (Foreman et al., 2003), endosperm development (Penfield et al., 2006), cellular vesicle cycle (Hao et al., 2014), seed after ripening (Muller et al., 2009), pollen tube growth, lipid microdomain polarization (Potocký et al., 2007; Hao et al., 2014; Nagano et al., 2016), plant-rhizobial symbiosis (Marino et al., 2011, 2012), and also stress responses as reviewed previously (Chang et al., 2016). The functional diversity was also observed in the wheat NOX gene family as discussed above. The results from our thorough coexpression analysis showed that different TaNOXs have different sets of coexpression genes against development stages, tissues, or stress conditions (Figure 9, Figures S4–S6, Table S5), implying the specific but complicated functions of NOX proteins in wheat development regulation and/or stress responses.

Corresponding to the developmental stages and tissue specificity, the coexpression genes with *TaNOXs* are mainly related to enzymatic glycosyl transfer, energy metabolism, substrate transport, cell wall synthesis, redox reaction, phosphorylation, and/or gene expression regulation (Figures S4, S5, Table S5), suggesting their diverse and vital function in wheat development. For example, *TaNOX2* is highly coexpressed with a kinase (casein kinase 1.5) and two cellulose synthases (CESA3 and CESA5) in all the tissues examined, implying that its function may be tightly related to the cell wall synthesis and protein phosphorylation. Casein kinase 1 (CK1) plays a crucial role in regulating growth and development via phosphorylating various substrates throughout the eukaryote kingdom. In *Arabidopsis*, two CK1s were found to regulate blue light signaling by phosphorylating cryptochrome 2 (Tan et al., 2013). CESA3 and CESA5 are two important proteins functioning in plant cell wall formation (Griffiths et al., 2015) and also in fiber cellulose production (Li et al., 2013). Several RLK family genes and a phytosulfokin receptor 1 gene were found to have significant coexpression with *TaNOX3* (Table S5), demonstrating that TaNOX3 might participate in a series of cellular signaling pathways. As reported earlier, RLKs play crucial roles in plant growth, development, and defence response by perceiving and transmitting extracellular or intracellular signals (Wu and Zhou, 2013). As the receptor of phytosulfokine (PSK), the phytosulfokin receptor 1 functions in cell growth, pollen tube guidance, and cell differentiation (Sauter, 2015). Several heavy metal transport-related proteins and DHHC-type zinc finger family proteins are highly coexpressed with TaNOX7 (Table S5), implying its functions in substrate transport and protein anchoring to the membrane. DHHC-type zinc finger family proteins were found to play crucial roles in protein palmitoylation for cell membrane anchoring (Mitchell et al., 2006). Besides being coexpressed with two CESA1-cellulose synthases, TaNOX8 also has high coexpression with a SCAMP family protein and two kinesin motor family proteins, indicating that it might not only participate in cell wall biosynthesis but also function in intracellular substance transport. SCAMP family proteins acting as carriers are responsible for the protein transport functioning to the cell surface in post-Golgi recycling pathways (Fernandez-Chacon et al., 2000), and kinesin family proteins unidirectionally transport various cargos including membranous organelles, protein complexes, and mRNA (Li et al., 2012). TaNOX12, however, is highly coexpressed with several glycosyl transfer-related proteins, demonstrating their roles in enzymatic glycosyl transfer. Moreover, two riboflavin synthase-like superfamily proteins have high coexpression with TaNOX12. Riboflavin synthases are responsible for the biosynthesis of vitamin B_2_, which is vital for the cellular redox balance (Fischer and Bacher, 2008). TaNOX13 is coexpressed with three CESA proteins and one CK1 protein, suggesting its functions in the cell wall formation and protein phosphorylation. It should be noticed that TaNOX12 has obvious coexpression with TaNOX2 and TaNOX8 at different development stages and in different tissues (Table S4), showing somewhat cross-talk between the function of different TaNOXs. All these results indicate the distinct but crossing functions of the TaNOXs in wheat growth and development.

The coexpression patterns of TaNOXs with other genes are greatly affected by different stresses, and different sets of genes were found to be highly coexpressed with the TaNOXs under stress conditions (Figure 9, Figure S6, Table S5), showing the vital roles of TaNOXs with complicated regulatory mechanisms during their functioning in stress tolerance. The genes that are highly coexpressed with TaNOXs under stress conditions are widely involved in stress signal transduction, cell wall formation, protein degradation, transcriptional regulations, and so on. Among the eight TaNOXs examined here, four genes including *TaNOX2, 3, 8*, and *13* have distinct coexpression with the genes encoding several protein degradation-related proteins including one ubiquitin-specific protease family C19-related protein, two ARM repeat superfamily proteins, one RING/U-box superfamily protein, and five COP1-interacting protein-related proteins (Figure S6, Table S5). All these coexpressed proteins function as E3 ubiquitin ligase or involve in ubiquitination of proteins (Zeng et al., 2008; Acosta et al., 2012; Xu et al., 2014), implying these TaNOXs might participate in protein degradation in wheat under stress conditions. Furthermore, a set of signal transduction-related genes were found to be highly coexpressed with TaNOXs, especially under stress conditions. At first, it was found that the genes encoding five protein kinase superfamily proteins are highly coexpressed with the TaNOXs, including TaNOX2, 7, and 13 (Table S5). These protein kinases, including an LRR family protein and two CC-NBS-LRR proteins, belong to receptor-like protein kinase (RLK) family, often functioning in signal transduction in plant cells, especially in the pathogen detection and defense response (Wu and Zhou, 2013). Moreover, it was observed that TaNOX3 is highly coexpressed with the genes encoding two BRASSINOSTEROID INSENSITIVE1 (BRI1) precursor kinases, and TaNOX7 is coexpressed with three PLC-like phosphodiesterases superfamily proteins (Figure 9, Figure S6, Table S5). BRI1 is a key component in brassinosteroid (BR) perception and signal transduction and has a broad impact on plant growth and development (Oh et al., 2012). Phospholipase C (PLC) is a class of membrane-associated enzymes playing a crucial role in signal transduction pathways (Singh et al., 2015). Our results obtained here have given much cues for the functions of TaNOXs and the possible working mechanism in stress tolerance but needs further investigation.

The coexpression of TaNOXs with several CESA proteins is another interesting event, especially under stress conditions. A total of nine CESA cellulose synthases were found to be highly coexpressed with the TaNOXs including TaNOX2, 8, and 13 (Table S5), indicating that these TaNOXs have important roles in cell wall formation both under normal and stress conditions. CESA cellulose synthases are the key enzymes for cellulose synthesis during the cell wall formation (Endler and Persson, 2011). It was also proposed that ROS, such as superoxide anion radical and hydrogen peroxide, function in cell wall loosing and stiffing, and in these processes, the NOX-mediated ROS production plays an important role (Karkonen and Kuchitsu, 2015). Furthermore, the ROS production also plays a key role in the process of cell wall remodeling under abiotic stresses (Tenhaken, 2015). All of these results suggest that some complicated regulatory mechanisms might be involved in the developmental regulation and stress response of TaNOXs in wheat.

Several other stress response-related genes were also found to be highly coexpressed with certain TaNOXs, further demonstrating the wide functions but complicated regulatory mechanisms of the TaNOXs during the plant development and stress responses. For instance, TaNOX3 is coexpressed with glutathione *S*-transferase tau 7, and TaNOX13 has coexpression with NAD kinase 1 (NADK1). Glutathione *S*-transferase (GST) is a multifunctional ROS scavenging enzyme that is involved in a variety of cellular processes. In *Arabidopsis*, a homolog of glutathione *S*-transferase tau 7 was found to be related to detoxification, secondary metabolism, and stress responses to *Botrytis cinerea* or *Pseudomonas syringae* (Fode et al., 2008). NADKs are the enzymes found in both prokaryotes and eukaryotes, generating pyridine nucleotide NADP(H) from substrates ATP and NAD(H). NADP(H) can be used as the electron donor to NOXs for generating ROS in plants (Turner et al., 2004). In *Arabidopsis*, NADK1 was found to be induced by environmental stresses and participated in plant pathogen interaction (Berrin et al., 2005). Moreover, the *TaNOXs* including *TaNOX7, 12*, and *13* also exhibit high coexpression with the genes encoding two *O*-fucosyltransferase family proteins, one starch synthase 2, and two melibiase family proteins, respectively, which all play important roles in glycometabolism. All these results suggest that the TaNOXs themselves or with their coexpression genes, synergistically or antagonistically, display pleiotropic functions in a wide range of processes of substance and energy metabolism, detoxification, substance transport, development regulation, and defence response, again implying the specific but complicated functions of the NOX genes in the wheat development and/or stress responses.

Taken together, wheat has multiple members of NOXs with diverse but vital functions in plant growth and development regulation and stress responses. Every member of TaNOXs has its specific expression pattern and function. Meanwhile, the functions of each wheat NOX and its regulatory mechanism in development regulation and defence response are still unclear. The interactions among TaNOXs and other genes are still barely reported in detail. The results obtained have provided a valuable foundation for further exploring the functions of NOX genes in wheat and in other plants as well.

## Author contributions

K-MC proposed concept. C-HH and P-QW collected data. C-HH, X-YW, and BY made the abiotic stress treatments. C-HH, X-YW, L-BY, T-TM, P-PZ, XW, W-TL, W-QL, and L-SM contributed for coexpression data. K-MC and C-HH wrote the manuscript.

### Conflict of interest statement

The authors declare that the research was conducted in the absence of any commercial or financial relationships that could be construed as a potential conflict of interest.
