# Supplementary material for: Genome-Wide Identification and Functional Analysis of NADPH Oxidase Family Genes in Wheat During Development and Environmental Stress Responses
Source: Front Plant Sci. 2018 Jul 23;9:906. doi: 10.3389/fpls.2018.00906 (PMC6065054; doi:10.3389/fpls.2018.00906)
Supplement: Supplementary file 1 [file Presentation_1.pdf]

frontiers

Supplementary Material

Genome-wide identification and functional analysis of NADPH oxidase family genes in wheat during development and environmental stress responses

Chun-Hong Hu[1, 2†], Xiao-Yong Wei[1†], Bo Yuan[1], Lin-Bo Yao[1], Tian-Tian Ma[1], Peng-Peng Zhang[1], Xiang Wang[1], Peng-Qi Wang[1], Wen-Ting Liu[1], Wen-Qiang Li[1], Lai-Sheng Meng[3], Kun-Ming Chen[1*]

[1], State Key Laboratory of Crop Stress Biology in Arid Areas, College of Life Sciences, Northwest A&F University, Yangling 712100, Shaanxi, P. R. China.
[2], Department of general biology, College of Life Science and Agronomy, Zhoukou Normal University, Zhoukou 466000, henan, P. R. China.
[3], The Key Laboratory of Biotechnology for Medicinal Plant of Jiangsu Province, School of Life Sciences, Jiangsu Normal University, Xuzhou 221116, Jiangsu, P. R. China.
[†], These authors contributed equally to this work.

*Correspondence author: Kunming Chen
State Key Laboratory of Crop Stress Biology in Arid Area/College of Life Sciences, Northwest A&F University, Yangling, Shaanxi, 712100, China.
Tel.: +86-29-8708-1178; Fax: +86-29-8709-2262.
Email: kunmingchen@nwsuaf.edu.cn; kunmingchen@163.com

E-mail addresses of authors: Chun-Hong Hu, ourcarrot@163.com; Xiao-Yong Wei, weixiaoyongshengwu@163.com; Bo Yuan, jax911911@gmail.com; Lin-Bo Yao, 675053332@qq.com; Tian-Tian Ma, 9447w@sina.com; Peng-Peng Zhang, 15256849763@163.com; Xiang Wang, wangxiang1342@126.com; Peng-Qi Wang, wangpengqi@nwafu.edu.cn; Wen-Ting Liu, lwt1635@yahoo.com; Wen-Qiang Li, wqli@nwsuaf.edu.cn; Lai-Sheng Meng, menglsh@jsnu.edu.cn; Kun-Ming Chen, kunmingchen@nwsuaf.edu.cn

1. Supplementary Figures and Tables

1.1 Supplementary Figures

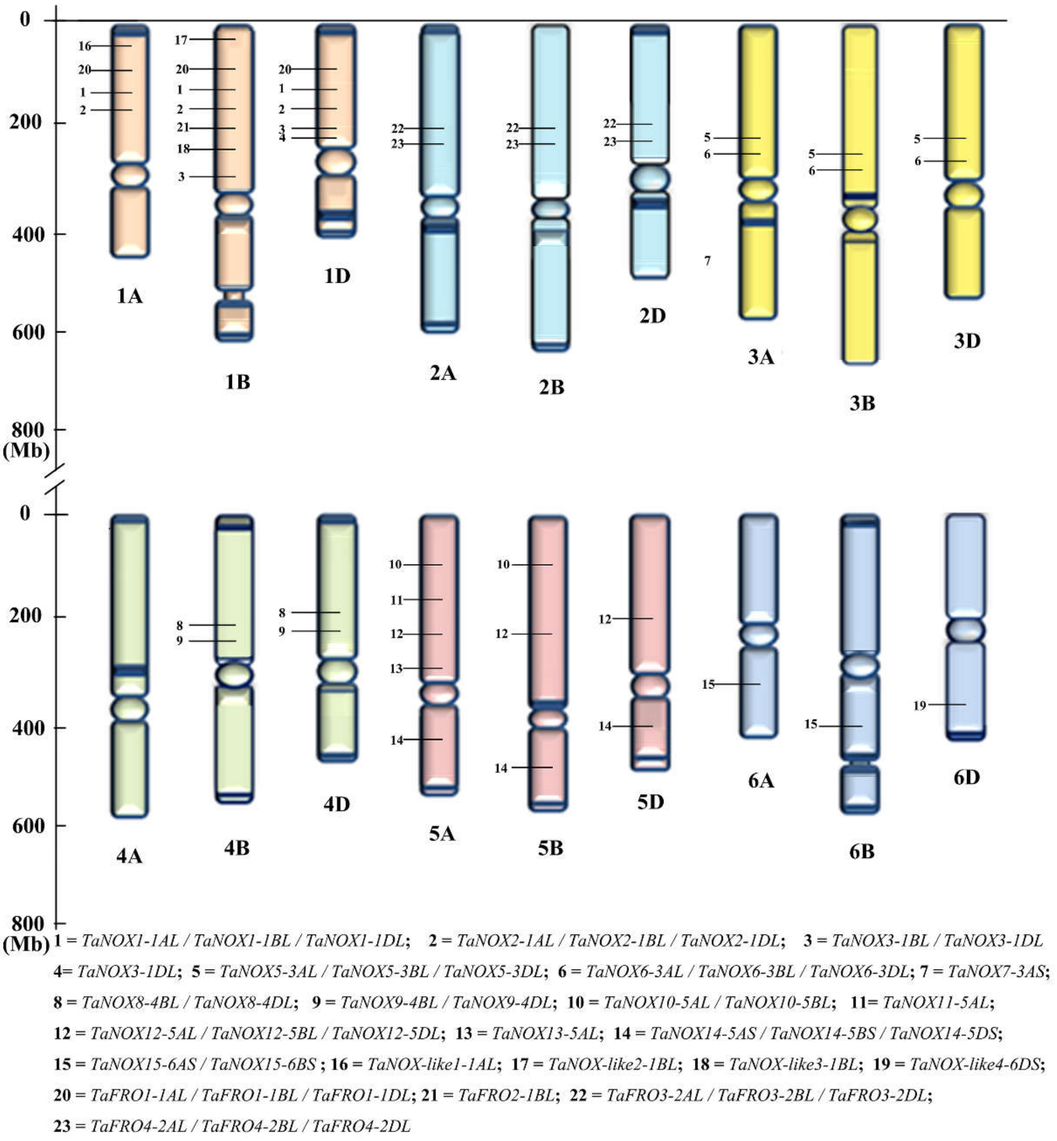

Figure S1. Chromosomal locations of wheat TaNOXs. The graph was drawn by Adobe_Photoshop_CS6 according to the information from scaffolds and Gene ID of each wheat NOX genes and the original sketchs were gotten from the WHEAT URGI website (https://wheat-urgi.versailles.inra.fr/Seq-Repository). TaNOXs positions are indicated by short horizontal lines and numbers.

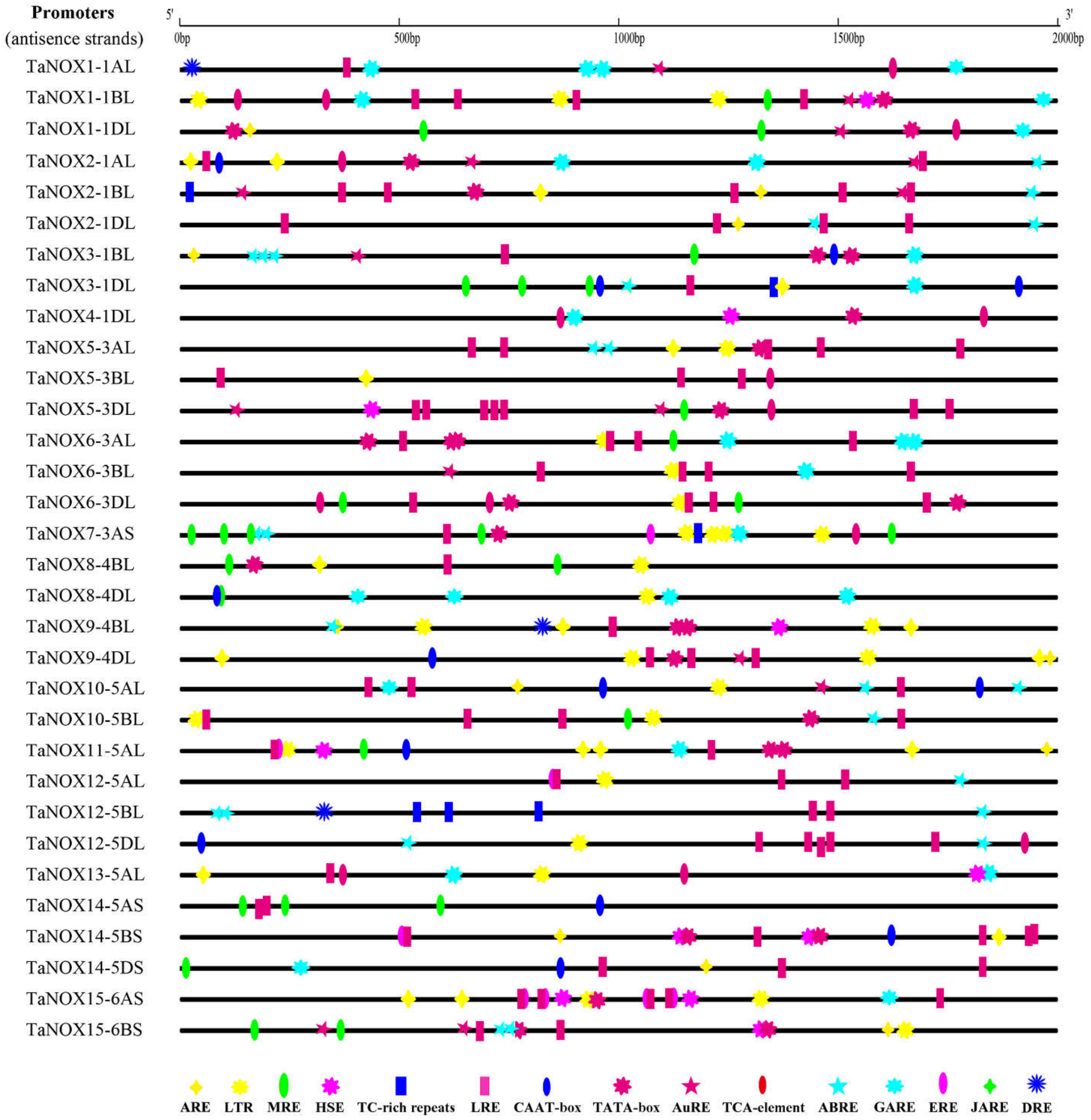

Figure S2. The cis-elements responding to abiotic-/biotic-stresses and hormone treatments in the antisense strands of the TaNOX promoters. The cis-elements are as follows. Anaerobic responsive element (ARE): ARE and GC-motif; LTR: low-temperature responsiveness; MYB responsive element (MRE): MBS and MRE; HSE: heat stress responsiveness; TC-rich repeats: defense and stress responsiveness; Light responsive element (LRE): G-box, SPI, MNF1,4cl-CMA2b,GT1-motif, ACE, AAC-motif; 3AF1-binding site and BoxI elements; CAAT-box: common cis-acting element in promoter and enhancer regions; TATA-box: core promoter element around -30 of transcription start; Auxin-responsive element (AuRE): AuXRR-core and TGA-element; TCA-element: salicylic acid (SA) responsiveness; Abscisic acid responsive element (ABRE): ABRE, motif IIb and CE3 elements; Gibberellins (GA)-responsive element (GARE): GARE, TATC-box and p-box; ERE: ethylene-responsive element; Methl jasmonic acid (MeJA) responsive element (JARE): TGACG-motif and CGTCA-motif.

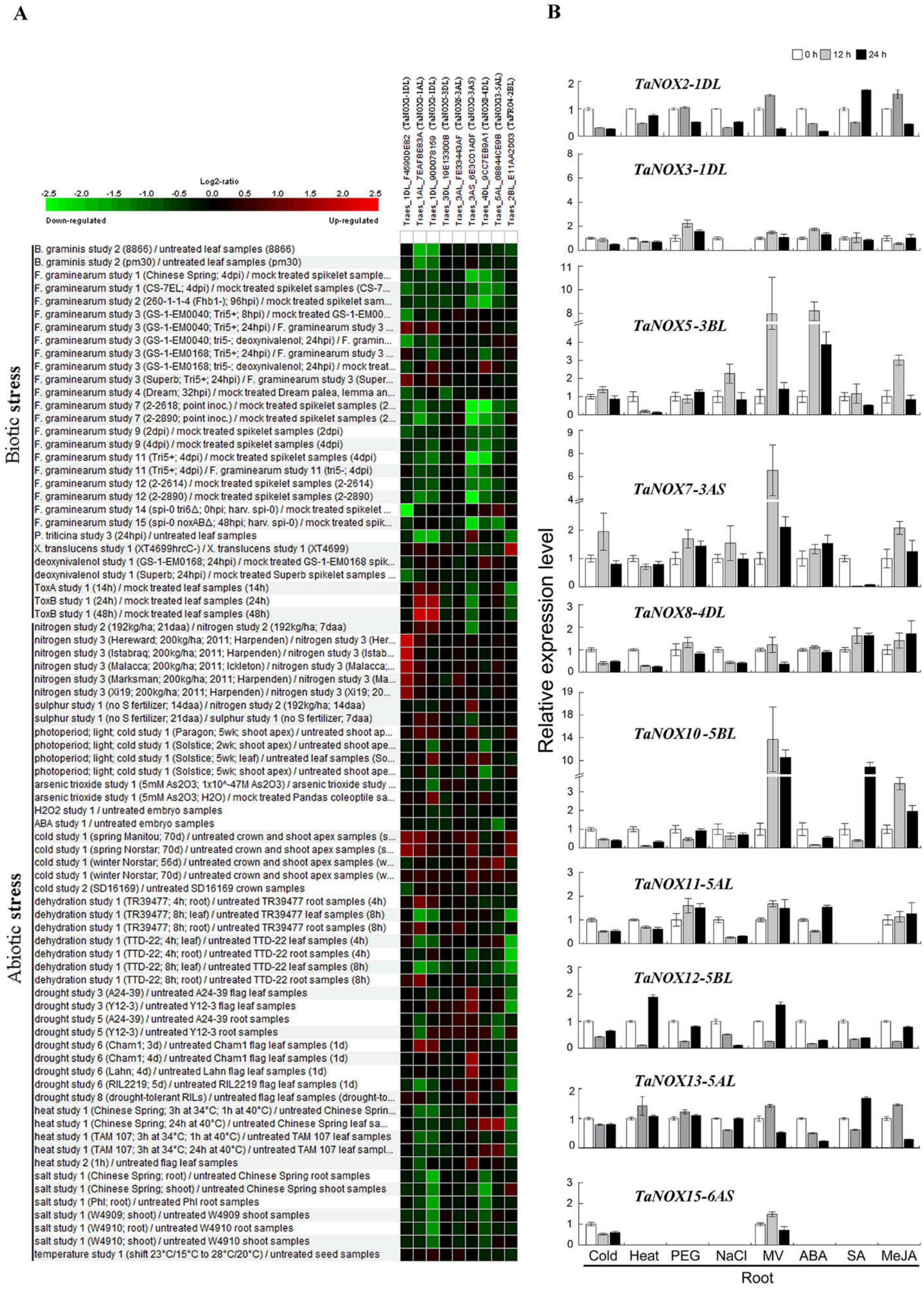

Figure S3. Inducible patterns of TaNOXs under different biotic and abiotic stresses. (A) The expression profiles obtained from the database of TA_AFFY_WHEAT-0 as reported by Genevestigator v3, illustrating different expression levels of TaNOXs under different biotic and abiotic stresses. Results were given as heat

maps in green/red coding that reflect relative signal values; where greener represents stronger down-regulated expression and redder represents stronger up-regulated expression. (B) The inducible expression patterns of wheat NOX family genes in roots under abiotic stresses and hormone treatments. Expression levels of the TaNOXs were assayed by qRT-PCR under cold (4°C), heat (40°C), 20% PEG6000, salt (200 mM NaCl), and oxidative (30 μM MV) stresses and by ABA (100 μM), SA (500 μM), MeJA (100 μM) hormone treatments. Two-week old seedlings were used for the analysis. Data are means ± SD (n = 3) and are representative of similar results from three independent experiments with three or four replicates for each experiment.

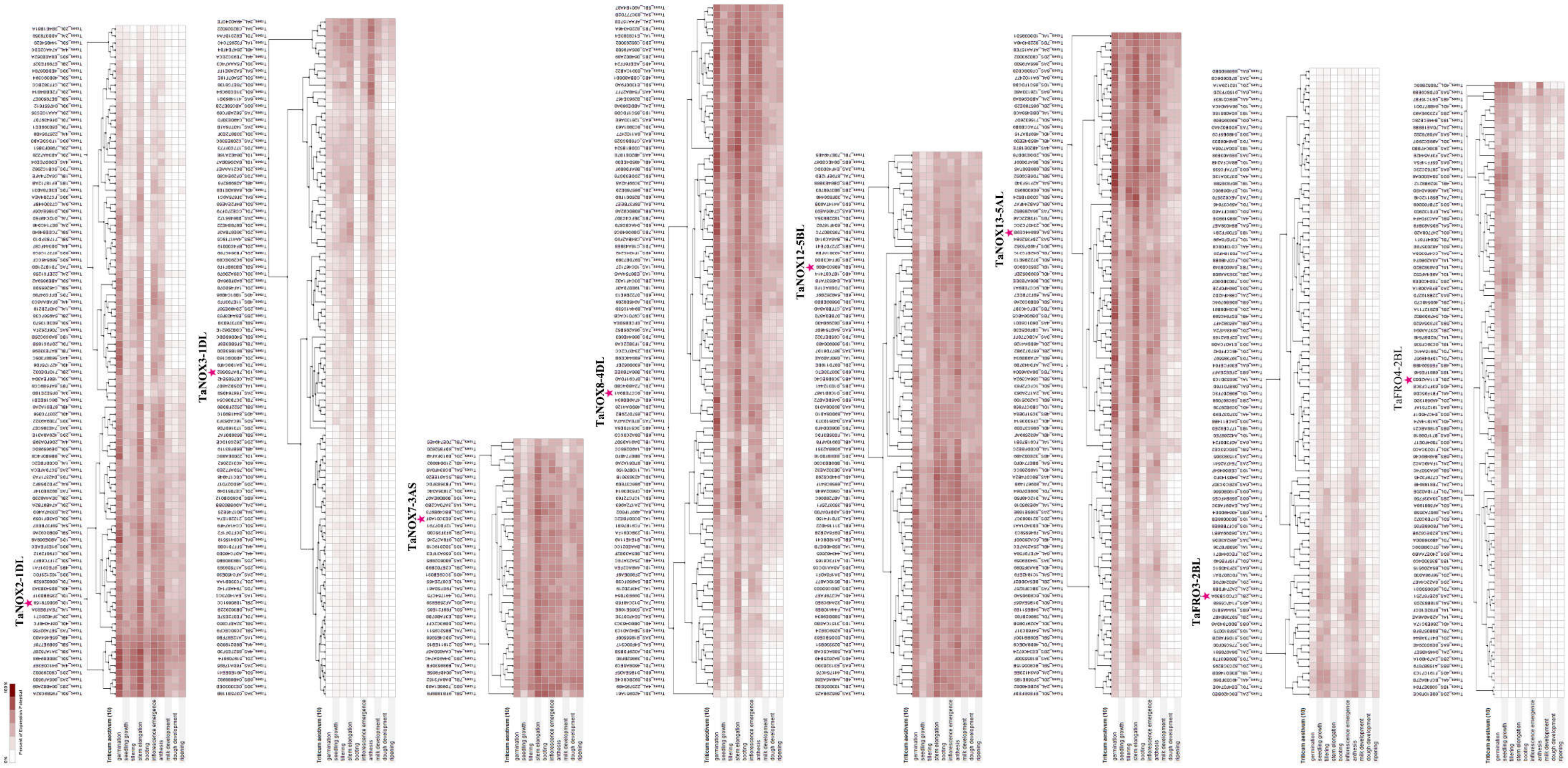

Figure S4. The coexpression patterns between wheat *TaNOX* genes and others at ten different developmental stages. The coexpression relationship between eight wheat *TaNOXs* and other genes were inferred from the database of Ta_mRNASeq_WHEAT_GL-0 with Genevestigator v3. The eight *TaNOX* genes including TaNOX2-1DL, TaNOX3-1DL, TaNOX7-3AS, TaNOX8-4DL, TaNOX12-5BL, TaNOX13-5AL, TaFRO3-2BL and TaFRO4-2BL, were selected for the analysis. The ten developmental stages include geinimation, sheeding growth, tillering, stem elongation, booting, inflorescence emergence, anthesis, milk development, dough debelopment and ripening. ★ : Repreasants the target gene among the eight *TaNOXs*.

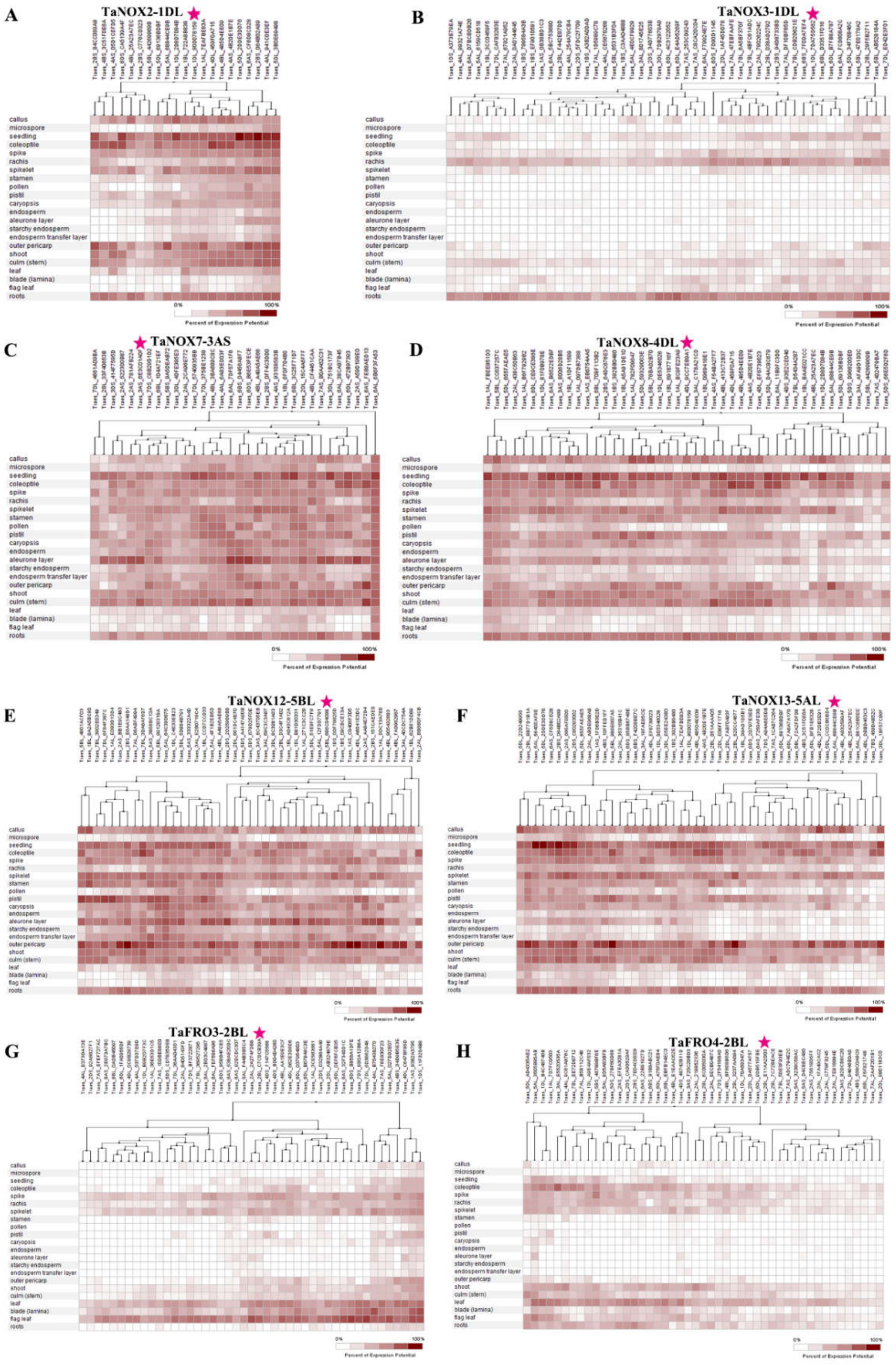

Figure S5. The coexpression patterns between wheat *TaNOX* genes and others in twenty two different tissues of wheat. The coexpression relationship between eight *TaNOXs* and other genes were inferred from the database of Ta_mRNASeq_WHEAT_GL-0 with Genevestigator v3. The eight TaNOX genes are the same to those in figure S4. The twenty two tissues include callus, microspore, coleoptile, spike, rachis, spikelet, stamen, pollen, pistil, caryopsis, endosperm, aleurone layer, ltarchy endosperm, endosperm transfer layer, outer pericarp, shoot, culm(stem), leaf, blade(lamina), flag leaf, roots. ★ : Repreasants the target gene among the eight *TaNOXs*.

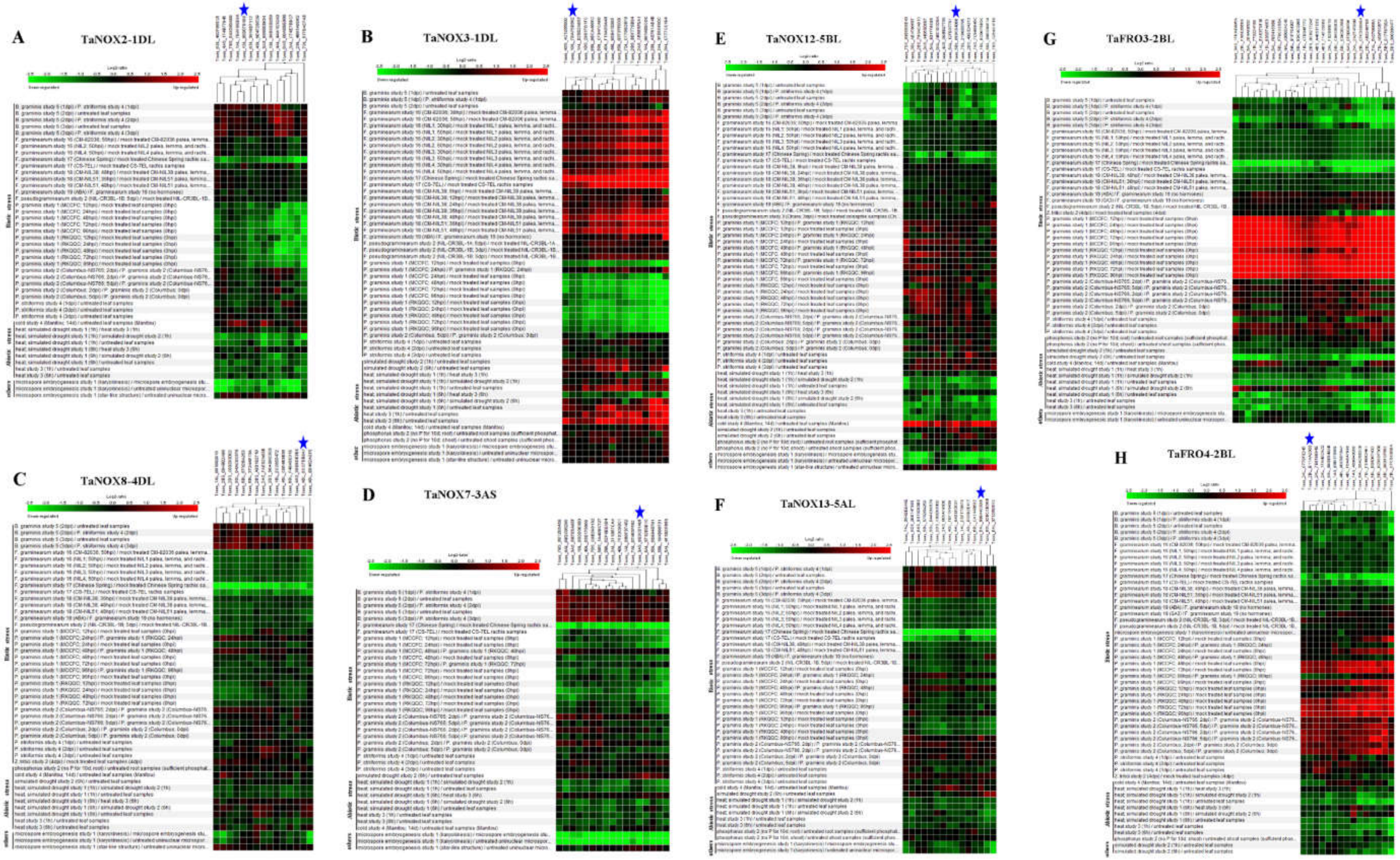

Figure S6. The coexpression relationship between wheat *TaNOX* genes and others under biotic and abiotic stresses. The response profiles between eight *TaNOXs* and other genes in wheat were inferred from the database of Ta_mRNASeq_WHEAT_GL-0 with Genevestigator v3. The eight TaNOX genes are the same to those in figure S4. ★ : Repreasants the target gene of the *TaNOXs*.

1.2 Supplementary Tables

Table S1. The detailed information about the members of wheat *NOXs* gene family

Nomenclature of wheat *NOX*			Genomic Location	Gene ID	Chr	Loc	TMH	A.A.	MW/Da	pI	Different Number of A.A.		
											A-B	B-D	A-D
TaNOXs	*TaNOX1*	*TaNOX1-1AL*	TGACv1_scaffold_002060_1AL:21147-25845	TRIAE_CS42_1AL_TGACv1_002060_AA0038250.1	1A	Plas	4	832	94214.27	8.92			
		TaNOX1-1BL	TGACv1_scaffold_030848_1BL:25006-29614	TRIAE_CS42_1BL_TGACv1_030848_AA0102150.1	1B	Plas	4	890	100557.77	9.16	74	20	69
		TaNOX1-1DL	TGACv1_scaffold_061459_1DL:54937-59575	TRIAE_CS42_1DL_TGACv1_061459_AA0195890.1	1D	Plas	4	888	100477.76	9.22			
	TaNOX2	*TaNOX2-1AL*	TGACv1_scaffold_002261_1AL:13467-22435	TRIAE_CS42_1AL_TGACv1_002261_AA0040140.2	1A	Plas	4	972	109103.56	9.42			
		TaNOX2-1BL	TGACv1_scaffold_031365_1BL:95301-103840	TRIAE_CS42_1BL_TGACv1_031365_AA0112470.1	1B	Plas	6	972	108913.98	9.23	68	22	67
		TaNOX2-1DL	TGACv1_scaffold_062127_1DL:37147-45975	TRIAE_CS42_1DL_TGACv1_062127_AA0209090.2	1D	Plas	4	968	108569.55	9.23			
	TaNOX3	*TaNOX3-1BL*	TGACv1_scaffold_034166_1BL:14946-24528	TRIAE_CS42_1BL_TGACv1_034166_AA0143700.2	1B	Plas	4	1145	130412.86	8.87		137	
		TaNOX3-1DL	TGACv1_scaffold_061195_1DL:74304-85785	TRIAE_CS42_1DL_TGACv1_061195_AA0188450.1	1D	Plas	4	1160	131928.72	8.88			
	TaNOX4	*TaNOX4-1DL*	TGACv1_scaffold_061195_1DL:100775-108485	TRIAE_CS42_1DL_TGACv1_061195_AA0188480.1	1D	Plas	6	999	110812.52	9.18			
	TaNOX5	*TaNOX5-3AL*	TGACv1_scaffold_195088_3AL:18969-23946	TRIAE_CS42_3AL_TGACv1_195088_AA0644550.1	3A	Plas	4	851	95748.79	9.44			
		TaNOX5-3BL	TGACv1_scaffold_225180_3B:6031-11525	TRIAE_CS42_3B_TGACv1_225180_AA0805890.2	3B	Plas	4	850	95660.72	9.47	9	12	10
		TaNOX5-3DL	TGACv1_scaffold_249608_3DL:46197-51065	TRIAE_CS42_3DL_TGACv1_249608_AA0852330.2	3D	Plas	4	847	95315.30	9.45			
	TaNOX6	*TaNOX6-3AL*	TGACv1_scaffold_195736_3AL:18346-27475	TRIAE_CS42_3AL_TGACv1_195736_AA0653240.3	3A	Plas	4	976	109814.33	9.42			
		TaNOX6-3BL	TGACv1_scaffold_221373_3B:62111-69519	TRIAE_CS42_3B_TGACv1_221373_AA0738650.2	3B	Plas	4	944	106663.66	9.26	49	11	43
		TaNOX6-3DL	TGACv1_scaffold_251733_3DL:24311-31685	TRIAE_CS42_3DL_TGACv1_251733_AA0884390.2	3D	Plas	6	946	106852.90	9.28			
	TaNOX7	*TaNOX7-3AS*	TGACv1_scaffold_211536_3AS:54051-73959	TRIAE_CS42_3AS_TGACv1_211536_AA0691290.1	3A	Plas	4	901	101630.76	9.35			
	TaNOX8	*TaNOX8-4BL*	TGACv1_scaffold_320896_4BL:55358-63622	TRIAE_CS42_4BL_TGACv1_320896_AA1051070.2	4B	Plas	6	847	95330.74	9.19		14	
		TaNOX8-4DL	TGACv1_scaffold_343050_4DL:40633-46817	TRIAE_CS42_4DL_TGACv1_343050_AA1128500.1	4D	Plas	6	841	94791.09	9.21			
	TaNOX9	*TaNOX9-4BL*	TGACv1_scaffold_321503_4BL:27846-36005	TRIAE_CS42_4BL_TGACv1_321503_AA1061470.3	4B	Plas	4	920	103696.93	9.34		10	
		TaNOX9-4DL	TGACv1_scaffold_343029_4DL:55967-62975	TRIAE_CS42_4DL_TGACv1_343029_AA1127810.3	4D	Plas	4	921	103724.99	9.37			
	TaNOX10	*TaNOX10-5AL*	TGACv1_scaffold_374312_5AL:121236-124735	TRIAE_CS42_5AL_TGACv1_374312_AA1196640.2	5A	Plas	4	831	93520.63	9.50	101		
		TaNOX10-5BL	TGACv1_scaffold_404354_5BL:132580-133920	BankIt2018780 Seq1MF166871(providedby gene bank)	5B	Plas	4	918	102796.57	9.24			
	TaNOX11	*TaNOX11-5AL*	TGACv1_scaffold_375026_5AL:12006-19009	TRIAE_CS42_5AL_TGACv1_375026_AA1214240.1	5A	Plas	4	919	103462.43	9.28			
		TaNOX12-5AL	TGACv1_scaffold_376348_5AL:15676-34722	TRIAE_CS42_5AL_TGACv1_376348_AA1235550.1	5A	Plas	4	989	110951.73	9.23			

Table S1. Continued

Nomenclature of wheat *NOX*		Genomic Location	Gene ID		Chr	Loc	TMH	A.A.	MW/Da	pI	Different Number of A.A.		
											A-B	B-D	A-D
TaNOXs		TaNOX12-5BL	TGACv1_scaffold_406190_5BL:21326-32065	TRIAE_CS42_5BL_TGACv1_406190_AA1342020.1	5B	Plas	4	985	110737.51	9.19			
		TaNOX12-5DL	TGACv1_scaffold_433213_5DL:35340-45258	TRIAE_CS42_5DL_TGACv1_433213_AA1405680.1	5D	Plas	4	994	111265.9	9.19			
	TaNOX13	TaNOX13-5AL	TGACv1_scaffold_377290_5AL:27991-34187	TRIAE_CS42_5AL_TGACv1_377290_AA1245640.1	5A	Plas	6	847	95219.60	9.18			
	TaNOX14	*TaNOX14-5AS*	TGACv1_scaffold_393979_5AS:10356-14798	TRIAE_CS42_5AS_TGACv1_393979_AA1277500.2	5A	Plas	4	877	98755.13	9.10			
		TaNOX14-5BS	TGACv1_scaffold_424084_5BS:25677-29885	TRIAE_CS42_5BS_TGACv1_424084_AA1386730.1	5B	Plas	4	873	98179.39	9.16	36	25	31
		TaNOX14-5DS	TGACv1_scaffold_458299_5DS:17176-21163	TRIAE_CS42_5DS_TGACv1_458299_AA1493220.1	5D	Plas	4	865	97360.53	9.11			
	TaNOX15	*TaNOX15-6AS*	TGACv1_scaffold_641235_U:99086-105234	TRIAE_CS42_U_TGACv1_641235_AA2089190.1	6A	Plas	4	956	106597.02	9.26		28	
		TaNOX15-6BS	TGACv1_scaffold_513624_6BS:121044-129225	TRIAE_CS42_6BS_TGACv1_513624_AA1646010.1	6B	Plas	4	957	106848.12	9.04			
TaNOX-likes	*TaNOX-like1*	*TaNOX-like1-1AL*	TGACv1_scaffold_001598_1AL:24546-36314	TRIAE_CS42_1AL_TGACv1_001598_AA0032710.1	1A	Plas	0	651	73338.61	6.11			
	TaNOX-like2	*TaNOX-like2-1BL*	TGACv1_scaffold_030295_1BL:132356-145070	TRIAE_CS42_1BL_TGACv1_030295_AA0085740.1	1B	Plas	5	675	76654.61	9.41			
	TaNOX-like3	*TaNOX-like3-1BL*	TGACv1_scaffold_032482_1BL:22766-37498	TRIAE_CS42_1BL_TGACv1_032482_AA0130380.1	1B	Plas	0	602	67747.99	6.02			
	TaNOX-like4	*TaNOX-like4-6DS*	TGACv1_scaffold_542557_6DS:1-2175	TRIAE_CS42_6DS_TGACv1_542557_AA1724240.1	6D	Plas	0	506	55792.32	8.50			
TaFROs	*TaFRO1*	*TaFRO1-1AL*	TGACv1_scaffold_003503_1AL:5146-11302	TRIAE_CS42_1AL_TGACv1_003503_AA0050090.1	1A	Plas	6	792	88760.15	8.52			
		TaFRO1-1BL	TGACv1_scaffold_030604_1BL:113286-118880	TRIAE_CS42_1BL_TGACv1_030604_AA0095460.1	1B	Plas	6	822	92043.96	8.34	74	24	70
		TaFRO1-1DL	TGACv1_scaffold_061711_1DL:15506-23338	TRIAE_CS42_1DL_TGACv1_061711_AA0202110.1	1D	Plas	6	823	91985.01	8.49			
	TaFRO2	*TaFRO2-1BL*	TGACv1_scaffold_031571_1BL:51686-62773	TRIAE_CS42_1BL_TGACv1_031571_AA0116350.2	1B	Plas	5	670	76888.19	9.44			
	TaFRO3	*TaFRO3-2AL*	TGACv1_scaffold_093270_2AL:96022-100945	TRIAE_CS42_2AL_TGACv1_093270_AA0276290.1	2A	Plas	11	748	82260.86	8.57			
		TaFRO3-2BL	TGACv1_scaffold_130340_2BL:64246-69130	TRIAE_CS42_2BL_TGACv1_130340_AA0409100.1	2B	Plas	11	806	88741.40	9.21	74	88	26
		TaFRO3-2DL	TGACv1_scaffold_159911_2DL:34457-39345	TRIAE_CS42_2DL_TGACv1_159911_AA0544270.1	2D	Plas	11	747	82323.05	8.54			
	TaFRO4	*TaFRO4-2AL*	TGACv1_scaffold_093999_2AL:25654-28885	TRIAE_CS42_2AL_TGACv1_093999_AA0290910.1	2A	Plas	8	776	84824.23	9.53			
		TaFRO4-2BL	TGACv1_scaffold_129792_2BL:24851-27935	TRIAE_CS42_2BL_TGACv1_129792_AA0396070.1	2B	Plas	8	723	79348.82	9.63	94	43	84
		TaFRO4-2DL	TGACv1_scaffold_160193_2DL:15929-19285	TRIAE_CS42_2DL_TGACv1_160193_AA0547970.1	2D	Plas	8	722	79038.66	9.59			

The data source of wheat from IWGSC (http://www.wheatgenome.org/), e!EnsemblPlants (http://plants.ensembl.org/index.html), ExPASy (http://web.expasy.org/protparam/), Plant-mPLoc (http://www.csbio.sjtu.edu.cn/bioinf/plant-multi/) and TMHMM v.2.0 (http://www.cbs.dtu.dk/services/TMHMM/). A.A.: number of amino acids; MW: molecular weight; pI: theoretical isoelectric point; Chr.: chromosome; Loc: the subcellular localization; TMH: the numbers of transmembrane helix.

Table S2. *NOXs* and *FROs* gene family members in eight plant species

Lineages	Species	Nomenclature *NOX* and *FRO*	Gene ID	A.A.
Monocots	*Triticum aestivum*	*TaNOX1-1BL*	TRIAE_CS42_1BL_TGACv1_030848_AA0102150.1	890
		TaNOX2-1AL	TRIAE_CS42_1AL_TGACv1_002261_AA0040140.2	972
		TaNOX3-1DL	TRIAE_CS42_1DL_TGACv1_061195_AA0188450.1	1160
		TaNOX4-1DL	TRIAE_CS42_1DL_TGACv1_061195_AA0188480.1	999
		TaNOX5-3AL	TRIAE_CS42_3AL_TGACv1_195088_AA0644550.1	851
		TaNOX6-3AL	TRIAE_CS42_3AL_TGACv1_195736_AA0653240.3	976
		TaNOX7-3AS	*TRIAE_CS42_3AS_TGACv1_211536_AA0691290.1*	901
		TaNOX8-4DL	*TRIAE_CS42_4DL_TGACv1_343050_AA1128500.1*	841
		TaNOX9-4DL	TRIAE_CS42_4DL_TGACv1_343029_AA1127810.3	921
		TaNOX10-5BL	BankIt2018780 Seq1MF166871(provided by gene bank)	918
		TaNOX11-5AL	TRIAE_CS42_5AL_TGACv1_375026_AA1214240.1	919
		TaNOX12-5DL	TRIAE_CS42_5DL_TGACv1_433213_AA1405680.1	994
		TaNOX13-5AL	TRIAE_CS42_5AL_TGACv1_377290_AA1245640.1	847
		TaNOX14-5AS	TRIAE_CS42_5AS_TGACv1_393979_AA1277500.2	877
		TaNOX15-6BS	TRIAE_CS42_6BS_TGACv1_513624_AA1646010.1	957
		TaNOX-like1-1AL	TRIAE_CS42_1AL_TGACv1_001598_AA0032710.1	651
		TaNOX-like2-1BL	TRIAE_CS42_1BL_TGACv1_030295_AA0085740.1	675
		TaNOX-like3-1BL	TRIAE_CS42_1BL_TGACv1_032482_AA0130380.1	602
		TaNOX-like4-6DS	TRIAE_CS42_6DS_TGACv1_542557_AA1724240.1	506
		TaFRO1-1DL	TRIAE_CS42_1DL_TGACv1_061711_AA0202110.1	823
		TaFRO2-1BL	TRIAE_CS42_1BL_TGACv1_031571_AA0116350.2	670
		TaFRO3-2BL	TRIAE_CS42_2BL_TGACv1_130340_AA0409100.1	806
		TaFRO4-2AL	TRIAE_CS42_2AL_TGACv1_093999_AA0290910.1	776
	Triticum urartu	*TuNOX1*	Triticum_urartu_TRIUR3_04873	705
		TuNOX2	Triticum_urartu_TRIUR3_10760	625
		TuNOX3	Triticum_urartu_TRIUR3_17466	731
		TuNOX4	Triticum_urartu_TRIUR3_18286	865
		TuNOX5	Triticum_urartu_TRIUR3_20633	624
		TuNOX6	Triticum_urartu_TRIUR3_20792	805
		TuNOX7	Triticum_urartu_TRIUR3_25647	843
		TuNOX8	Triticum_urartu_TRIUR3_26076	606
		TuFRO1	Triticum_urartu_TRIUR3_14484	707
		TuFRO2	Triticum_urartu_TRIUR3_21493	839
		TuFRO3	Triticum_urartu_TRIUR3_31975	1019
	Aegilops tauschii	*AetNOX1*	Aegilops_tauschii_F775_05767	946
		AetNOX2	Aegilops_tauschii_F775_06338	731
		AetNOX3	Aegilops_tauschii_F775_08558	797
		AetNOX4	Aegilops_tauschii_F775_11876	669
		AetNOX5	Aegilops_tauschii_F775_16752	726
		AetNOX6	Aegilops_tauschii_F775_18726	1091
		AetNOX7	Aegilops_tauschii_F775_21135	600
		AetNOX8	Aegilops_tauschii_F775_28509	726

Table S2. Continued

Lineages	Species	Nomenclature *NOX* and *FRO*	Gene ID	A.A.
Monocots		*AetFRO1*	Aegilops_tauschii_F775_08531	639
		AetNOX9	Aegilops_tauschii_F775_30688	717
		AetNOX10	Aegilops_tauschii_F775_52502	1176
		AetFRO2	Aegilops_tauschii_F775_09519	707
		AetFRO3	Aegilops_tauschii_F775_10160	707
	Hordeum vulgare	*HvNOX1*	Hordeum_vulgare_MLOC_5021	845
		HvNOX2	Hordeum_vulgare_MLOC_5633	862
		HvNOX3	Hordeum_vulgare_MLOC_11469	832
		HvNOX4	Hordeum_vulgare_MLOC_51084	807
		HvNOX5	Hordeum_vulgare_MLOC_54087	726
		HvNOX6	Hordeum_vulgare_MLOC_73746	720
		HvNOX7	Hordeum_vulgare_MLOC_81745	906
		HvFRO1	Hordeum_vulgare_MLOC_63739	602
		HvFRO2	Hordeum_vulgare_MLOC_77866	515
	Brachypodium distachyon	*BdNOX1*	Bradi2g12790.2 (BD2G12790)	901
		BdNOX2	Bradi2g19090.1 (BD2G19090)	989
		BdNOX3	Bradi2g22820.1 (BD2G22820)	835
		BdNOX4	Bradi2g49040.1 (BD2G49040)	943
		BdNOX5	Bradi2g54240.1 (BD2G54240)	845
		BdNOX6	Bradi3g37530.1 (BD3G37530)	987
		BdNOX7	Bradi4g05540.1 (BD4G05540)	856
		BdNOX8	Bradi4g17020.1 (BD4G17020)	924
		BdNOX9	Bradi4g31130.1 (BD4G31130	991
		BdFRO1	Bradi5g11147.1 (BD5G11147)	758
		BdFRO2	Bradi5g19150.1 (BD5G19150)	735
	Oryza sativa	*OsNOX1*	LOC_Os01g25820.1 (Os01g0360200)	880
		OsNOX2	LOC_Os01g53294.1 (Os01g0734466)	727
		OsNOX3	LOC_Os01g61880.1 (Os01g0835500)	843
		OsNOX4	LOC_Os05g38980.1 (Os05g0465800)	819
		OsNOX5	LOC_Os05g45210.1 (Os05g0528000)	951
		OsNOX6	LOC_Os08g35210.1 (Os08g0453700)	1033
		OsNOX7	LOC_Os09g26660.1 (Os09g0438000)	1007
		OsNOX8	LOC_Os11g33120.1 (Os11g0537400)	936
		OsNOX9	LOC_Os12g35610.1 (Os12g0541300)	892
		OsFRO1	LOC_Os04g36720.1 (Os04g0444800)	758
		OsFRO2	LOC_Os04g48930.1 (Os04g0578600)	525
	Zea mays	*ZmNOX1*	GRMZM2G448185_T01 (ZM01G37420)	1009
		ZmNOX2	GRMZM2G065144_T01 (ZM02G42940)	948
		ZmNOX3	GRMZM2G358619_T01 (ZM02G42960)	976
		ZmNOX4	GRMZM2G037993_T01 (ZM02G42980)	882
		ZmNOX5	GRMZM2G034896_T01 (ZM03G12260)	921
		ZmNOX6	GRMZM2G401179_T01 (ZM03G14570)	842
		ZmNOX7	GRMZM2G426953_T01 (ZM03G27690)	852

Table S2. Continued

Lineages	Species	Nomenclature *NOX* and *FRO*	Gene ID	A.A.
		ZmNOX8	GRMZM2G138152_T01 (ZM03G32550)	943
		ZmNOX9	GRMZM2G441541_T03 (ZM04G30500)	932
		ZmNOX10	GRMZM2G043435_T01 (ZM06G29020)	948
		ZmNOX11	GRMZM2G147966_T01 (ZM07G15130/40)	999
		ZmNOX12	GRMZM2G022547_T02 (ZM08G08220)	897
		ZmNOX13	GRMZM2G323731_T01 (ZM08G08900)	822
		ZmNOX14	GRMZM2G300965_T01 (ZM10G04920)	931
		ZmFRO1	GRMZM2G089291_T01 (ZM01G20940)	624
		ZmFRO2	GRMZM2G068557_T01 (ZM02G14250)	760
Eudicots	*Arabidopsis thaliana*	*AtRBOHA*	AT5G07390.1	902
		AtRBOHB	AT1G09090.1	843
		AtRBOHC	AT5G51060.1	905
		AtRBOHD	AT5G47910.1	921
		AtRBOHE	AT1G19230.1	934
		AtRBOHF	AT1G64060.1	944
		AtRBOHG	AT4G25090.1	849
		AtRBOHH	AT5G60010.1	886
		AtRBOHI	AT4G11230.1	941
		AtRBOHJ	AT3G45810.1	912
		AtFRO1	AT1G01590.1	704
		AtFRO2	AT1G01580.1	725
		AtFRO3	AT1G23020.1	717
		AtFRO4	AT5G23980.1	699
		AtFRO5	AT5G23990.1	657
		AtFRO6	AT5G49730.1	738
		AtFRO7	AT5G49740.1	747
		AtFRO8	AT5G50160.1	728

Table S3. The identical percentage of amino acids sequences between homologue gene pairs

Subgroups	Homologoue gene pairs		Identical percentage / %
I	*TaNOX11-5AL*	*TaNOX9-4DL*	98.7
	TaNOX9-4DL	*BdNOX8*	89
	TaNOX10-5BL	*AetNOX4*	98.9
	TaNOX3-1DL	*AetNOX6*	98.7
	TaNOX4-1DL	*OsNOX8*	74.3
	TaNOX15-6BS	*TaNOX-like4*	97.9
II	*TaNOX12-5DL*	*OsNOX7*	93.3%
	TaNOX12-5DL	*BdNOX9*	95.1%
	TaNOX12-5DL	*ZmNOX11*	95.6%
	TaNOX6-3AL	*TaNOX2-1AL*	86.6
	TaNOX6-3AL	*ZmNOX8*	90.8
	TaNOX2-1AL	*OsNOX2*	88.4
III	*TaNOX13-5AL*	*TaNOX8-4DL*	97.5
	TaNOX13-5AL	*ZmNOX12*	88.8
	TaNOX8-4DL	*ZmNOX12*	87.5
	TaNOX14-5AS	*OsNOX9*	86.3
	TaNOX7-3AS	*OsNOX1*	87.3
	TaNOX7-3AS	*ZmNOX5*	81.2
IV	*TaNOX-like1*	*AetNOX7*	52.1
	TaNOX-like3	*AetNOX7*	59.8
V	*TaNOX5-3AL*	*OsNOX3*	90.8
	TaNOX5-3AL	*AtRbohG*	64.3
	TaNOX1-1BL	*AtRbohG*	65.9
VI	*TaFRO3-2BL*	*OsFRO1*	76.4

Table S4. Rates of non-synonymous (Ka) and synonymous (Ks) nucleotide substitutions and their ratios (ω) for protein-coding nucleotide sequence of paralogous and orthologous gene pairs of NOX family genes in four species*

Gene pairs	Ka	Ks	ω (Ka/ Ks)	Divergence time (Mya)
***Triticum aestivum* VS. *Triticum aestivum* (AABBDD, 2n = 6×= 42)**				
TaNOX4-1DL VS. *TaNOX6-3AL*	2.1470	1.7086	1.256584338	6.1166
TaNOX9-4DL VS. *TaNOX10-5BL*	1.2268	0.5256	2.334094368	3.1663
TaNOX1-1BL VS. *TaNOX12-5DL*	2.3250	1.3319	1.745626549	6.2533
TaNOX8-4DL VS. *TaNOX13-5AL*	0.0073	0.1414	0.051626591	0.1159
***Triticum aestivum* (AABBDD, 2n = 6×= 42) VS. *Aegilops tauschii* (DD, 2n = 2×= 14)**				
TaNOX1-1BL VS. *AetNOX3*	0.0087	0.0589	0.14770798	0.0606
TaNOX3-1DL VS. *AetNOX6*	0.0084	0.0054	1.555555556	0.0231
TaNOX5-3AL VS. *AetNOX2*	0.0054	0.0613	0.088091354	0.0551
TaNOX10-5BL VS. *AetNOX4*	0.0200	0.0692	0.289017341	0.0955
TaNOX4-1DL VS. *AetNOX10*	0.0090	0.0074	1.216216216	0.0259
TaNOX7-3AS VS. *AetNOX1*	0.0053	0.0424	0.125	0.0420
***Triticum aestivum* (AABBDD, 2n = 6×= 42) VS. *Triticum urartu* (AA, 2n = 2×= 14)**				
TaNOX1-1BL VS. *TuNOX3*	0.0083	0.0757	0.109643329	0.0710
TaNOX2-1AL VS. *TuNOX6*	0.0275	0.0421	0.653206651	0.0927
TaNOX3-1DL VS. *TuNOX4*	0.1697	0.3622	0.468525676	0.6466
TaNOX11-5AL VS. *TuNOX5*	0.0492	0.0784	0.62755102	0.1683
TaNOX12-5DL VS. *TuNOX8*	0.0135	0.0810	0.166666667	0.0893
TaNOX13-5AL VS. *TuNOX7*	0.0005	0.0069	0.072463768	0.0060
TaNOX14-5AS VS. *TuNOX1*	0.0019	0.0039	0.487179487	0.0071
TaNOX15-6BS VS. *TuNOX2*	0.0071	0.0948	0.074894515	0.0859
***Triticum aestivum* (AABBDD, 2n = 6×= 42) VS. *Hordeum vulgare* (AA, 2n = 2×= 14)**				
TaNOX1-1BL VS. *HvNOX3*	0.0388	0.1388	0.279539	0.1853
TaNOX5-3AL VS. *HvNOX4*	0.0032	0.0818	0.03912	0.0650
TaNOX6-3AL VS. *HvNOX5*	0.0072	0.1728	0.041667	0.1384
TaNOX7-3AS VS. *HvNOX6*	0.0196	0.1017	0.192724	0.1163
TaNOX14-5AS VS. *HvNOX2*	0.0175	0.0821	0.213155	0.1003

* The non-synonymous (Ka) and synonymous (Ks) were estimated for the paralogous and orthologous gene pairs of NOX family genes in four species, *Triticum aestivum, Aegilops tauschii, Triticum urartu* and *Hordeum vulgare*, by using the bioinformatics software pamlX1.2 based on the aligned CDS sequences of each orthologous and paralogous gene pairs which the terminator codons were removed. Divergence time (T, million years ago, Mya) for each paralogous gene pair was calculated using the average Ks of λ substitutions per synonymous sites per year as T =Ks/2λ ($\lambda = 6.5 \times 10^{-9}$).

Table S5. The detailed information of coexpression genes with the 8 *TaNOX* genes

Wheat *NOX* genes	Coexpression genes (probe ID)	PCC	Functions of the coexpression genes
**Coexpression genes at different developmental stages**			
Traes_1DL_90d078159	Traes_1AL_7EAFBe83A	1	Respiratory burst oxidase protein(TaNOX2-1AL)
(*TaNOX2-1DL*)	Traes_6DS_1FDCDEAD0	0.98	Endoglucanase: ATP binding,InterProprotein kinase activity
	Traes_1AL_0C0DFBE2C	0.98	S-locus lectin protein kinase family protein: ATP bindingprotein,serine/threonine kinase activity
	Traes_3DL_1B8FEA3D4	0.97	DNAse-like superfamily protein
	Traes_2BL_3EA4A823D	0.97	O-fucosyltransferase family protein
Traes_1DL_7DA75D562	Traes_7DL_9A1D8C4F8	0.99	Leucine-rich repeat protein kinase family protein: ATP binding protein,serine/threonine kinase activity
(*TaNOX3-1DL*)	Traes_3AS_0375B118B	0.98	S-locus lectin protein kinase family protein: ATP bindingprotein,serine/threonine kinase activity
	Traes_1AL_D25D2A8D7	0.98	Respiratory burst oxidase homologue
	Traes_4DS_8441B901C	098	2-oxoglutatate (2OG) and Fe(II)-dependent oxygenase: metal ion binding, oxidoreductase activity
	Traes_2DL_1AF45DD78	0.98	Phenylalanine ammonia-lyase 4
Traes_3AS_6E3C01A0F	Traes_5AL_DC634F0A5	0.96	Heavy metal transport detoxification superfamily protein
(*TaNOX7-3AS*)	Traes_2AL_8DFB52620	0.96	Leucine-rich repeat protein kinase family protein: ATP binding, protein tyrosine kinase activity
	Traes_2DL_BBC49B973	0.95	Kinesin motor domain containing protein
	Traes_5BL_5CA61EE26	0.94	DHHC-type zinc finger family protein
	Traes_2DL_9F8AC7245	0.94	CLIP-associated protein
Traes_4DL_9CC7EB9A1	Traes_6AL_B1E4E11A9	0.97	Zinc finger (C3HC4-type RING finger) family protein
(*TaNOX8-4DL*)	Traes_1AS_10C467127	0.97	CESA1-cellulose synthase
	Traes_6AL_9A1102477	0.97	SCAMP family protein: protein transport, secretory carrier membrane protein
	Traes_1DS_65C1FDCD8	0.96	CESA1- cellulose synthase
	Traes_4DL_1A9D268CC	0.96	Remorin family protein
Traes_5BL_686C04088	Traes_5DS_A4147A90B	0.97	Galactosyltransferase family protein
(*TaNOX12-5BL*)	Traes_5BS_9A5DEAB72	0.95	Galactosyltransferase family protein

Table S5. Continued

Wheat *NOX* genes	Coexpression genes (probe ID)	PCC	Functions of the coexpression genes
	Traes_5BL_C66D2A645	0.94	Transport protein particle(TRAPP) component, pollen tube development, response to abscisic acid
	Traes_3DL_D5689EBBD	0.93	SCD1
	Traes_5DL_9269C8E24	0.92	RING/FYVE/PHD zinc finger superfamily protein
Traes_5AL_68844CE9B	Traes_4BL_25A23A7EC	0.97	Respiratory burst oxidase homolog B(TaNOX8BL)
(*TaNOX13-5AL*)	Traes_3AL_2A172A963	0.97	CTP synthase family protein: catalyzes the ATP-dependent amination of UTP to CTP with either L-glutamine or ammonia as the source of nitrogen
	Traes_4BS_3C51F0E6A	0.96	Hydroxyproline-rich glycoprotein family protein
	Traes_2AL_6A34123EE	0.96	Arogenate dehydratase 6
	Traes_2BL_BE5A3DB2F	0.96	Glutaredoxin family protein: electron carrier activity; protein disulfide oxidoreductase activity; cell redox homeostasis
Traes_2BL_C7CDCB39A	Traes_2DL_AB324879E/	0.99	Ferric reduction oxidase (TaFRO3DL)
(*TaFRO3-2BL*)	Traes_2AL_2A274FDB8/	0.99	Ferric reduction oxidase (TaFRO3AL)
	Traes_1BS_9DA08515E	0.98	HXXXD-type acyl-transferase family protein
	Traes_1DS_6CF7BB6E8	0.98	Concanavalin A-like lectin protein kinase family protein: ATP binding,protein kinase activity
	Traes_1DS_BD30088EB	0.98	Protein kinase family protein: ATP binding,protein kinase activity
Traes_2BL_E11AA2D03	Traes_4DL_5479D9B02	0.96	Mechanosensitive channel of small conductance-like: Atransmembrane transport
(*TaFRO4-2BL*)	Traes_6AS_28752C23C	0.96	Chloride channel: voltage-gated chloride channel activity
	Traes_1BS_6681FE545	0.95	Cysteine-rich repeat secretory protein 55 precursor
	Traes_1AL_A2DA8A6AE	0.95	Cytokinin dehydrogenase precursor, putative, expressed
	Traes_5DL_AF588196A	0.95	UDP-glucosyl transferase 73C2
**Coexpression genes in different tissues**			
Traes_1DL_90d078159	Traes_1AL_7EAFBE83A	0.95	Respiratory burst oxidase Protein F(TaNOX2AL)
(*TaNOX2-1DL*)	Traes_5AL_68844CE9B	0.92	Respiratory burst oxidase homologue B(TaNOX13AL)

Table S5. Continued

Wheat *NOX* genes	Coexpression genes (probe ID)	PCC	Functions of the coexpression genes
	Traes_4BS_3C51F0E6A	0.91	Hydroxyproline-rich glycoprotein family protein
	Traes_5DL_69136BDBF	0.90	CK1_CaseinKinase_1.5-CK1 includes the casein kin: ATP binding, protein kinase activity
	Traes_1BL_722A8B838	0.90	Respiratory burst oxidase Protein F (TaNOX2BL)
	Traes_4BL_25A23A7EC	0.90	Respiratory burst oxidase homologue B(TaNOX8BL)
	Traes_5DL_3B0E69498	0.90	CESA5-cellulose synthase
	Traes_4AL_941C0E3EF	0.98	CESA3-cellulose synthase
Traes_1DL_7DA75D562 (*TaNOX3-1DL*)	Traes_4AL_99321A74E	0.96	LRR and NB-ARC domains-containing resistance protein: leucine rich repeats(LRRs) play an important role in plant growth, development, disease resistance and defense mechanism
	Traes_7BL_6A5BF370F	0.96	Leucine-rich repeat protein kinase family protein
	Traes_2BL_038AD2792	0.95	Hydrolase, NUDIX family, domain containing protein
	Traes_6BL_D3351F016	0.95	Phytosulfokin receptor 1
	Traes_6AL_D7BCBDB26	0.94	Phytosulfokin receptor 1
	Traes_4AL_CE5670268	0.94	RNG/U-box superfamily protein
	Traes_2DL_1AF45DD78	0.92	Phenylalanine ammonia-lyase 4
	Traes_7BL_4BFC61ADC	0.92	Leucine-rich repeat protein kinase family protein
	Traes_6AL_FCD808A2C	0.92	Phytosulfokin receptor 1
Traes_3AS_6E3C01A0F (*TaNOX7-3AS*)	Traes_6DS_B6E53FECE	0.90	Single hybrid motif superfamily protein
	Traes_2AS_781AFB224	0.89	DHHC-type zinc finger family protein: have palmitoyltransferase activity, (posseses a positive control function in tiller of rice)
	Traes_6BS_04456A8F7	0.84	Single hybrid motif superfamily protein
	Traes_7DL_5740935BB	0.84	Endomembrane protein 70 protein family
	Traes_7AS_B0AA62C31	0.83	P-loop containing nucleoside triphosphate hydrolases: GTPase activity
	Traes_5BS_18894	0.79	Pectin lyase-like superfamily protein

Table S5. Continued

Wheat *NOX* genes	Coexpression genes (probe ID)	PCC	Functions of the coexpression genes
	Traes_5DS_A4147	0.79	Galactosyltransferase family protein
	Traes_5AL_4F1B2	0.79	PLC-like phosphodiesterases superfamily protein
	Traes_5AL_53530	0.79	E3 ubiquitin- protein ligase HERC2
	Traes_4BS_98118	0.78	Heavy metal transport/detoxification superfamily protein
Traes_4DL_9CC7EB9A1	Traes_3AL_C178A21CD	0.95	Glyoxalase II 3
(*TaNOX8-4DL*)	Traes_1AL_097DB7389	0.94	Kinesin motor family protein
	Traes_7AS_402479BA7	0.93	UDP-Glycosyltransferase superfamily protein: metal ion binding,methyltransferase activity
	Traes_1BL_A1DF11589	0.92	Kinesin motor family protein: microtubule motor activity,ATP binding
	Traes_1DL_81F0B978E	0.91	3-hydroxyacyl-CoA dehydrogenase, putative, expressed
	Traes_5DL_D4AC8C679	0.90	COPI-interacting protein-related protein
	Traes_5AL_68844CE9B	0.88	Respiratory burst oxidase homologue B(TaNOX13AL)
	Traes_5DS_D966200E0	0.87	Plecin-related protein
Traes_5BL_686C04088	Traes_5AL_12FDD7791	0.96	Riboflavin synthase-like superfamily protein
(*TaNOX12-5BL*)	Traes_2BS_E6AA149D1	0.92	Glycoprotein membrane precursor GPI-anchored
	Traes_1DS_0DF78825D	0.91	GDSL-like Lipase/Acylhydrolase superfamily protein: hydrolase activity, acting on ester bonds
	Traes_2BS_151CAEDED	0.91	Heavy metal transport/detoxification superfamily protein
	Traes_1BL_A043C812A	0.89	Glycosyltransferase family 61 protein
	Traes_5DL_51E9FC7F9	0.88	Riboflavin synthase-like superfamily protein
	Traes_5DS_679D25F0E	0.87	PLC-like phosphodiesterases superfamily protein
	Traes_4BL_A49A5AE66	0.86	Glucuronidase
	Traes_2BL_D3C4F1A02	0.84	Ubiquitin-specific protease family C19-related protein
Traes_5AL_68844CE9B	Traes_4BL_A632599AF	0.95	Respiratory burst oxidase homologue D (TaNOX8BL)
(*TaNOX13-5AL*)	Traes_4BL_25A23A7EC	0.95	Respiratory burst oxidase homologue B (TaNOX8BL)

Table S5. Continued

Wheat *NOX* genes	Coexpression genes (probe ID)	PCC	Functions of the coexpression genes
	Traes_1AL_7EAFBE83A	0.94	Respiratory burst oxidase protein F (TaNOX2AL)
	Traes_6DS_85BB674BE	0.94	IQ calmodulin-binding motif family protein
	Traes_5DL_69136BDBF	0.92	CK1_CaseinKinase_1.5-CK1 includes the casein kin: ATP binding, protein kinase activity
	Traes_4BS_3C51F0E6A	0.91	Hydroxyproline-rich glycoprotein family protein
	Traes_5DL_CC0CB6BD4	0.91	Heavy metal transport/detoxification superfamily protein
	Traes_6AS_CF6D8CD28	0.90	CESA1- cellulose synthase
Traes_2BL_C7CDCB39A	Traes_2DL_AB324879E	0.99	Ferric reduction oxidase 7 (TaFRO3DL)
(*TaFRO3-2BL*)	Traes_2AL_2A274FDB8	0.99	Ferric reduction oxidase 6 (TaFRO3AL)
	Traes_6DL_0EF6FCB35	0.95	Leucine-rich repeat protein kinase family protein: ATP binding, protein,serine/threonine kinase activity
	Traes_6DS_B856A1DFE	0.95	Cyclase family protein: arylformamidase activity,tryptophan catabolic process to kynurenine
	Traes_4BL_AA1E9E3CF	0.95	BTBN5-Bric-a-Brac, Tramtrack, Broad Complex BTB d
Traes_2BL_E11AA2D03	Traes_2AL_1FA49CAC2	0.96	Ferric reduction oxidase 4 (TaFRO4AL): oxidoreductase activity
(*TaFRO4-2BL*)	Traes_2AL_C779F3245	0.94	Ferric reduction oxidase 2
	Traes_2DS_CAD0529AF	0.93	EF hand family protein: calcium ion binding
	Traes_2AL_7E818894E	0.92	Ferric reduction oxidase 1:oxidoreductase activity（TaFRO4-2AL）
	Traes_2BS_7E04C0EE9	0.92	Calcium-binding EF-hand family protein: calcium ion binding
**Coexpression genes under different stresses**			
Traes_1DL_90d078159	Traes_1AL_7EAFBE83A	0.90	Respiratory burst oxidase protein(TaNOX2AL)
(*TaNOX2-1DL*)	Traes_3AL_C74E7BDC7	0.77	S-adenosyl-L-methionine-dependent methyltransferase: methyltransferase activity
	Traes_1BL_39BCEB359	0.76	ATP synthase alpha/beta family protein: Produces ATP from ADP in the presence of a proton gradient across the membrane
	Traes_7DL_517D4C7AE	0.75	Protein kinase superfamily protein: ATP binding, protein serine/threonine kinase activity
	Traes_7BS_6A3290A89	0.74	Selenoprotein O, putative, expressed

Table S5. Continued

Wheat *NOX* genes	Coexpression genes (probe ID)	PCC	Functions of the coexpression genes
	Traes_4AS_174B77945	0.74	SAC domain proteins
	Traes_6DL_492F66EC5	0.74	Diacylglycerol kinase 7
	Traes_2BL_4B61A6D62	0.74	Ubiquitin-specific protease family C19-related protein
	Traes_6DL_3916D7112	0.73	ARM repeat superfamily protein
Traes_1DL_7DA75D562	Traes_5BL_CF34FCA90	0.93	Respiratory burst oxidase homologue D
(*TaNOX3-1DL*)	Traes_1AL_D25D2A8D7	0.92	Respiratory burst oxidase homologue D (TaNOX1AL)
	Traes_4DL_F13AD3A4B	0.91	NADPH/respiratory burst oxidase protein D
	Traes_2AS_CE5E5F0A1	0.90	Brassinosteroid Insensitive 1 precursor kinase
	Traes_4BL_8D8410395	0.90	Respiratory burst oxidase homologue D
	Traes_2BL_AE761AE4B	0.89	Brassinosteroid Insensitive 1 precursor kinase
	Traes_2AL_3FE5DF00C	0.88	Alternative oxidase 1A
	Traes_6BL_8ECA4665C	0.87	ANTH (anthocyanin)
	Traes_3AL_C777CC194	0.87	Glutathione S-transferase tau 7
	Traes_5BL_83737603B*	0.87	RING/U-box superfamily protein: alternative oxidase activity, Binds 2 ions per subuni
Traes_3AS_6E3C01A0F	Traes_5BL_194BD8F31	0.91	PLC-like phosphodiesterases superfamily protein: phosphoric diester hydrolase activity
(*TaNOX7-3AS*)	Traes_7AL_07460F582	0.91	Disease resistance protein (CC-NBS-LRR) family protein
	Traes_5DL_5D984B791	0.88	PLC-like phosphodiesterases superfamily protein: phosphoric diester hydrolase activity
	Traes_4BL_7FD3C9BC1	0.88	O-fucosyltransgerase family protein
	Traes_5AL_4F1B2EB5D	0.88	PLC-like phosphodiesterases superfamily protein: phosphoric diester hydrolase activity
	Traes_4DL_53D175966	0.87	LRR family protein
	Traes_6AL_3712E0E1C	0.87	Melibiase family protein
	Traes_3AS_5B7E5A95F	0.87	FAD/NAD(P)-binding oxidoreductase
	Traes_7DS_C8B29D1D2	0.87	ARG1-like 1; heat shock protein

Table S5. Continued

Wheat *NOX* genes	Coexpression genes (probe ID)	PCC	Functions of the coexpression genes
Traes_4DL_9CC7EB9A1	Traes_2DS_C80293002	0.84	CESA8-cellusose synthase
(*TaNOX8-4DL*)	Traes_1AL_881882915	0.83	Myosin heavy chain-related protein
	Traes_1BL_2CC655472	0.82	Retrotransposon protein, putative, ty1-copia subclass
	Traes_2BS_064B02A89	0.82	CESA8-cellusose synthase
	Traes_2DS_D6AD8E3ED	0.81	GDP-fucose protein-O-fucosyltransferase2
	Traes_5BL_3724A613A	0.80	COPI interacting protein-related
	Traes_5DL_D4AC8C679	0.80	COPI interacting protein-related
	Traes_5BL_5732BA252	0.80	COPI interacting protein-related
	Traes_2BS_A90182710	0.80	ARM repeat superfamily protein
Traes_5BL_686C04088	Traes_6AL_AE01DC0EA	0.75	Starch synthase 2 (projected from AT3G01180):glycogen (starch) synthase activity
(*TaNOX12-5BL*)	Traes_4AS_D9BB27735	0.75	Expressed protein (projected from LOC_Os12g21789): hydrolase activity, hydrolyzing O-glycosyl compounds
	Traes_3DL_AE478655F	0.74	Alpha/beta-Hydrolases superfamily protein
	Traes_7AS_FC99B645C	0.74	CRAL/TRIO domain containing protein
	Traes_2BS_40E324D13	0.74	Aquaporin protein: transporter activity
	Traes_5AL_12FDD7791	0.74	Riboflavin synthase-like superfamily protein
	Traes_5BL_D88C59519	0.72	NADP-dependent oxidoreductase
	Traes_2BS_764ACA81C	0.72	4-alpha-glucanotransferase
	Traes_7BS_C25A31FF8	0.71	Cytochrome p450 superfamily protein
Traes_5AL_68844CE9B	Traes_6DL_32BCCED8B	0.79	Melibiase family protein: catalase activity
(*TaNOX13-5AL*)	Traes_2DS_C80293002	0.77	CESA8-cellulose synthase
	Traes_5DL_D4AC8C679	0.77	COPI interacting protein-related
	Traes_1AL_EEFF7B5F8	0.77	NAD kinase 1: NAD+ kinase activity, NAD metabolic process, NADP biosynthetic process
	Traes_5BL_5732BA252	0.77	COPI interacting protein-related

Table S5. Continued

Wheat *NOX* genes	Coexpression genes (probe ID)	PCC	Functions of the coexpression genes
	Traes_6DL_32BCCED8B	0.79	Melibiase family protein
	Traes_2AS_665AF9500	0.76	CESA8-cellulose synthase
	Traes_7AL_354EEE44E	0.76	OsIAA21-Auxin-responsive Aux/IAA gene family member
	Traes_5DS_268147383	0.74	plant specific transcription factor YABBY family protein
	Traes_6BL_CA144B632	0.74	NB-ARC domain containing disease resistance protein
Traes_2BL_C7CDCB39A (*TaFRO3-2BL*)	Traes_2AL_2A274FDB8	0.97	Ferric reduction oxidase 6 (TaFRO3AL)
	Traes_2DL_AB324879E	0.96	Ferric reduction oxidase 7 (TaFRO3DL)
	Traes_2BL_FD6E26509	0.93	Pathogenesis-related BetvI family protein: defense response, response to biotic stimulus
	Traes_1BL_C7D34F77C	0.93	Disease resistance protein (CC-NBS-LRR class) family protein: ADP binding
	Traes_4AL_A0565AC4C	0.92	RNA polymerase sigma factor, putative: transcription factor activity, sequence-specific DNA binding
Traes_2BL_E11AA2D03 (*TaFRO4-2BL*)	Traes_7DL_FE4B62461	0.87	Major facilitator superfamily protein: transporter activity
	Traes_2AL_C779F3245	0.86	Ferric reduction oxidase 2
	Traes_5BL_D20CD7D08	0.84	Double Clp-N motif-containing P-loop nucleoside triph
	Traes_2AL_1FA49CAC2	0.84	Ferric reduction oxidase 4 (TaFRO4AL)
	Traes_6DL_88DFCF552	0.84	C2H2-type zinc finger family protein: nucleic acid binding

PCC: Pearson correlation coefficient

Table S6. The primers used for qRT-PCR, gene clone, and vector construction in this study.

Gene Name	Primer Sequences	Introduction (bp)
**qRT-PCR for analysis of tissue and development expression level**		
TaNOX2(1DL)	Forward:5' AAGAGCCCGATCCGAAAAAT 3'	242
	Reverse:5' GTGTTGCGGCATACAGGTAGG 3'	
TaNOX3(1DL)	Forward:5'GCCTTCTTCAAGGTTTGCGAGC3'	116
	Reverse:5'GCCACGTACTTTAGTCGACCTC3'	
TaNOX5(3BL)	Forward:5'TGTTGCGAGGCACAAGTTACTTC3'	271
	Reverse:5'CTGCCTATCTCAGAACCGTGTA3'	
TaNOX7(3AS)	Forward:5' ATATTCATGCGAGCGCATT3'	275
	Reverse:5' AGAAGACTGTCCTAAGCCGAG 3'	
TaNOX8(4DL)	Forward:5'CAGGAGAACTGGAAGCGCATA3'	186
	Reverse:5'GTTGAATACAGCTCGGTTACGGT3'	
TaNOX10(5BL)	Forward:5'AACTGGCGCAAAGTCTACAAGGA3'	289
	Reverse:5'ATCGATGAAACTCTTGACCCAACT3'	
TaNOX11(5AL)	Forward:5'ACTTCCTCTACCTCACACACAA3'	222
	Reverse:5'CGCAGTTGACGAAGATGTAC3'	
TaNOX12(5BL)	Forward:5'ACCATAATGAGTGCATGAAGCCT3'	179
	Reverse:5'GGTCCGAAACCAACCCTATCTC3'	
TaNOX13(5AL)	Forward:5'GAGGAGAACTGGAAGCGCATAT3'	159
	Reverse:5'GAATTTCAGGGTCTCGGCG3'	
TaNOX15(6AS)	Forward:5'TGCCCAGCAGAGCTTCATTTGG3'	227
	Reverse:5'GGTCTCCATTGATAGGTCCATCA3'	
TaACTIN (AB181991.1)	Forward:5'GAAGTGCTTTTGAAGAGTCGGT3'	207
	Reverse:5'TTATTTCATACAGCAGGCAAGC3'	
TaGAPDH	Forward:5'TTAGACTTGCGAAGCCAGCA3'	81
(A0A1D6ATH8)	Reverse:5'AAATGCCCTTGAGGTTTCCC3'	
**QRT-PCR for expression level analysis of coexpression gene**		
TaNOX2(1DL)	Forward:5' AAGAGCCCGATCCGAAAAAT 3'	242
	Reverse:5' GTGTTGCGGCATACAGGTAGG 3'	
TaNOX3(1DL)	Forward:5'AGCAAGAATCCCTGGAGTGGT3'	273
	Reverse:5'CTCCGACACGTTTTCTTTGATTT3'	
TaNOX7(3AS)	Forward:5' ATATTCATGCGAGCGCATT3'	275
	Reverse:5' AGAAGACTGTCCTAAGCCGAG 3'	
TaNOX8(4DL)	Forward:5'CAGGAGAACTGGAAGCGCATA3'	105
	Reverse:5'GTTGAATACAGCTCGGTTACGGT3'	
TaNOX12(5BL)	Forward:5'ACCATAATGAGTGCATGAAGCCT3'	179
	Reverse:5'GGTCCGAAACCAACCCTATCTC3'	
Traes_1BL_39BCEB359	Forward:5'GATAAGATGACGGTTGCTCGC3'	82
	Reverse:5'CACCAGTGAAAACTTCGGCA3'	
Traes_3AS_8355038D4	Forward:5'GGTTCTTGCCTTCGGCTATGT3'	114
	Reverse:5'TCACGGGTGCAGAAGTATGAATAT3'	
Traes_4AS_174B77945	Forward:5'TGGTTACTACACGGTCAGCAAAAG3'	214
	Reverse:5'CGCTTTCACCAAGGCTACCA3'	

Traes_1AL_881A0ECDC	Forward:5'TGCCACCTACCCAGAGATTGA3'	245
	Reverse:5'ACTCCTAGCTGCTTCCGTTTTC3'	
Traes_2AS_CE5E5F0A1	Forward:5'TTTAGTTCAGTTGGGTGTTCGTTC3'	212
	Reverse:5'TGCCTGCCAAATAAATCAGTGTA3'	
Traes_3AL_C777CC194	Forward:5'GGACTACAACGCCGACGAAG3'	165
	Reverse:5'GAGGCATCACACGGGACCACT3'	
Traes_5BL_194BD8F31	Forward:5'TTCCTCCTCTCGCTCCACG3'	220

Table S6. Continued

Gene Name	Primer Sequences	Introduction (bp)
	Reverse:5'GGGTCACCAGCCATGAGTATCT3'	
Traes_6AL_3712E0E1C	Forward:5'TCCAGCAAGGAAGCAGTAGGTA3'	89
	Reverse:5'AGGTTGAAGAATGCGAGGTAGA3'	
Traes_2BS_A90182710	Forward:5'AGGTGGTAGTTCCCCGCAAG3'	211
	Reverse:5'GCCTTTGCGTCTTTGTTTCTATT3'	
Traes_5DL_D4AC8C679	Forward:5'AGATTCGCAAAACTGTCTTACGC3'	129
	Reverse:5'TCAAGCGTGAAGCACCAAAA3'	
Traes_5BL_5732BA252	Forward:5'AATAATGACGCGGATATGTTCG3'	180
	Reverse:5'CATCTGCCTGGCCCTAATTC3'	
Traes_7BS_C25A31FF8	Forward:5'ACTTCGTGGCGAGAATGGTG3'	154
	Reverse:5'TTTTGCGTGGGAAATGGTTAG3'	
Traes_2BS_40E324D13	Forward:5'CGTCGCCACCTTTCTGTCTAA3'	151
	Reverse:5'ATCATTCGGGCTTGAGGACTC3'	
Traes_6AL_AE01DC0EA	Forward:5'AAAGTGTGGAGTTCTCCGGTTG3'	165
	Reverse:5'TCTTCTTGCCAGAGCCTTCG3'	
**Promoter clone of *TaNOXS***		
TaNOX2-1AL	Forward:5'TCCCGCACAAATGGACAGCA3'	2285
	Reverse:5'ACCCCCCGCATCTTCCCTT3'	
TaNOX3-DL	Forward:5'GGGCATTTATTTCAACCAGCAC3'	1949
	Reverse:5'AGCAGAACCGAAGAAGCAGTAGT3'	
TaNOX7-3AS	Forward:5'TACTTCATCCGTTGTTCCGCACAA3'	2045
	Reverse:5'CTTTATTTACCTCTCAGGGCAATT3'	
TaNOX8-4DL	Forward:5'AACAACACGGACGGACGAAAG3'	2187
	Reverse:5'CCCTGTTTGAGCCTTCTAAGTTCC3'	
TaNOX10-5BL	Forward:5'TACTCGGCTATGTAGGTTGGATGT3'	1813
	Reverse:5'GGCCGGCCGCCGGCTCCAGCTAGA3'	
TaNOX12-5BL	Forward:5'GTTTGTGGCTACGGTTGGATGC3'	2200
	Reverse:5'CTCGTTGTCGGAGGTGTCGG3'	
TaNOX13-5AL	Forward:5'GCATCGGCATCACTGGCACG3'	2298
	Reverse:5'CCAGCTTCAATGTCAGCCATGTCC3'	
**Vector construction for PGL3-Promters::Rluc-35S::Fluc**		
TaNOX2-1AL	Forward:5'GGGGTACCTCCCGCACAAATGGACAGCAAGAG3'	2290
	Reverse:5'GGACTAGTCTTCCCTTGGGCAGAGCAGTCCGT3'	
TaNOX3-1DL	Forward:5'GGGGTACCGGGCATTTATTTCAACCAGCACTGAAAC3'	1977

	Reverse:5'TCCCCCGGGCGGGTAGTAGCAGAACCGAAGAAGCAGT3'	
TaNOX7-3AS	Forward:5'CGGAATTCTACTTCATCCGTTGTTCCGCACAA3'	2063
	Reverse:5'AAAACTGCAGCTTTATTTACCTCTCAGGGCAATT3'	
TaNOX8-4DL	Forward:5'GGGGTACCAACAACACGGACGGACGAAAGGCC3'	2109
	Reverse:5'GGACTAGTCTCCTTCTTCTTTTACCTAACCCC3'	
TaNOX10-5BL	Forward:5'TACCGAGCTCGAATTCTACTCGGCTATGTAGGTTGGAT GT3'	1852
	Reverse:5'GTCATGGTGDNGCTAGCCTGCAGGGCCGGCCGCCGGCT CCAGCTAGA3'	

Table S6. Continued

Gene Name	Primer Sequences	Introduction (bp)
TaNOX12-5BL	Forward:5'GGGGTACCGTTTGTGGCTACGGTTGGATGCCC3'	2108
	Reverse:5'GGACTAGTGGCCGCTCCCCGCGCCCCGCCCCG3'	
TaNOX13-5AL	Forward:5'CGGAATTCGCATCGGCATCACTGGCACGTTTG3'	2294
	Reverse:5'AAAACTGCAGGTCCTTCTTCTTTTTACCTAACC3'	
**Gene clone for TaNOXS**		
TaNOX7-3AS	Forward:5'GGCCATGGCTGATATCGGATCC3'	2760
	Reverse:5'TGGTGGTGGTGGTGCTCGAGTGC3'	
TaNOX10-5BL	Forward:5'TCTCATCACCATCTCCTCCTCCACCTACT3'	3277
	Reverse:5'TAACGGTCATCCTTCTTTCTTCTTCCTCT3'	
TaFRO4-2BL	Forward:5' GAGTGTCTGGAGTGACCAAATACG3'	2392
	Reverse:5' AGAAATGGTGATAGTCATAGAAACGA3'	
TaNOX-like4	Forward:5' TTTCGTGGACGGAGAAGATAGCA3'	1585
	Reverse: 5' GTACTCCCCCGGGCTCGAC3'	
**Vector construction for PTF486-35S:: TaNOXS: eGFP**		
TaNOX7-3AS	Forward:5'CGACTCTAGAGGATCCATGGAGATGCCTGATATTGAAGCTGGC3'	2732
	Reverse:5'TGCTCACCATGGATCCGAAGTTCTCCTTGTGAAACTCAAACTTG3'	
TaNOX10-5BL	Forward:5'GTCTACCATCAGCCCAGAGGATCCATGCATAACTACGGAGGCGG3'	2786
	Reverse:5'TGCTCACCATGGATCCGAAATTCTCCTTGTGGAACTCGAAT3'	
TaFRO4-2BL	Forward:5'GTCTACCATCAGCCCAGAGGATCCATGGACAATCGTACTCTTGGCAAAG3′	2209
	Reverse:5'TGCTCACCATGGATCCCCAGGTGAAGCTTATAGACTGGAAATG3'	
TaNOX-like4	Forward:5'GTCTACCATCAGCCCAGAGGATCCATGTGGACGCCGTCGCGAGG3'	1558
	Reverse:5'TGCTCACCATGGATCCCCGGTACTCCCCCGGGCTCGACGC3'	

2. Supplementary information S1 for experimental program

Sequence retrieval and identification of the NOX gene family in wheat

We retrieved the potential sequences of NOX family members including TaNOXs and TaFROs in wheat from IWGSC (http://www.wheatgenome.org/, last accessed May 25, 2017), NCBI (https://www.ncbi.nlm.nih.gov/, last accessed May 25, 2017), and e! Ensembl Plants (http://plants.ensembl.org/index.html, last accessed May 25, 2017), with *Arabidopsis* and rice NOX sequences as queries. Then we identified each NOX member by predicting the conserved domains in EMBL-EBI (http://pfam.xfam.org/search#tabview=tab1) and SMART (http://smart.embl-heidelberg.de/) websites. For further information, we analyzed the physicochemical parameters such as the molecular

weight (MW) and isoelectric point (pI) of the wheat NOX and FRO candidates with the ExPASY Compute pI/Mw Program (http://web.expasy.org/compute_pi/), predicted the subcellular localization with Plant-mPLoc (http://www.csbio.sjtu.edu.cn/bioinf/plant-multi/) and the numbers of transmembrane helix with TMHMM v2.0 (http://www.cbs.dtu.dk/services/TMHMM/), and performed amino acid sequence alignment by the software of BioEdit v7.0.5 and DNAMAN 6.0.

Sequence alignment and gene structure analysis

The un-rooted maximum-likelihood phylogenetic tree of wheat NOX and FRO family members was constructed by alignment the complete protein sequences with MEGA 6.06, in which the bootstrap was set to 1,000 replicates. The logos of domain organization were generated by submitting the full-length amino acid sequences to EMBL-EBI or SMART websites, and were amended with Adobe_Photoshop_CS6. The four conserved domain motifs, namely NADPH_Ox, Ferric_reduct, FAD_binding_8 and NAD_binding_6 in each NOX sequence, were generated by submitting the full-length amino acid sequences to MEME Suite (http://meme-suite.org/). These domains were used to perform multiple alignment analysis. The bits score means information content of each position in the amino acid sequence.

Phylogenetic relationships of NOX and FRO gene families in wheat and seven other plant species

Multiple sequence alignments and phylogenetic relationship analysis of NOX and FRO gene families were performed using the maximum-likelihood method with MEGA 6.06 based on the full-length protein sequences from eight plant species. Due to the fact that, the wheat NOX homologues assigned as the same number in gene denomination are similarity in gene structure and protein sizes each other, we selected the biggest ones (including 23 homologues) from those as the representations for phylogenetic analysis. All the amino acid sequences used here, including 23 from *Triticum aestivum*, 11 from *Triticum urartu*, 11 from *Brachypodium distachyon*, 9 from *Hordeum vulgare*, 13 from *Aegilops tauschii*, 16 from Zea mays, 11 from Oryza sativa, and 18 from *Arabidopsis thaliana*, were gotten from the e! Ensembl Plants website (http://plants.ensembl.org/index.html), the Arabidopsis Information Resource website (http://www.arabidopsis.org/), the Rice Genome Annotation Project website (http://rice.plantbiology.msu.edu/index.shtml), the URGI website (https://wheat-urgi.versailles.inra.fr/Seq-Repository) and the NCBI website (https://www.ncbi.nlm.nih.gov/), etc. To further analyze the phylogenetic relationships of NOX family genes, non-synonymous (Ka) and synonymous (Ks) in paralogous and/or orthologous gene pairs from four species, namely *Triticum aestivum*, *Aegilops tauschii*, *Triticum urartu* and *Hordeum vulgare*, were estimated using the bioinformatics software PAMLX 1.2 based on the aligned CDS sequences of each paralogous gene pairs with the terminator codons removed. Divergence time (T, million years ago, Mya) for each orthologous or paralogous gene pairs were estimated using the average Ks of λ substitutions per synonymous sites per year.

Subcellular localization analysis

Analysis of the subcellular location of wheat NOXs were performed in rice protoplasts using transient transformation systems with some modifications (Zhang et al., 2011). The full-length open reading frame (ORF) of *TaNOX7*, *TaNOX10, TaFRO4 and TaNOX-like4* genes were used for the construction of vectors. The merged sequences of TaNOX7::eGFP, TaNOX10::eGFP, TaFRO::eGFP and *TaNOX-like4*::eGFP were under the control of the constitutive 2×35S promoter (cauliflower mosaic virus: CaMV) in pTF486 vector, which were transformed into rice protoplasts. After 16 h incubation with gentle shaking (45 rpm) at 23°C in darkness, the transformed protoplasts were observed with a confocal microscope (A1R, Nikon, Tokyo, Japan). At least three monoclones were sent for sequencing upon gene cloning and vector construction for each examined gene.

Prediction and functional analysis of cis-regulatory elements

The 2,000 bp promoter sequences of TaNOXs (named as TaNOX-pros) selected from the genomic DNA sequences upstream of the transcriptional start sites were used to analyze the cis-acting elements. The cis-acting elements existing in the promoters were predicted by the databases: PlantCARE (http://bioinformatics.psb.ugent.be/webtools/plantcare/html/) (Lescot et al., 2002), PLACE (http://www.dna.affrc.go.jp/PLACE/). Each cis-element logo and position were drawn manually according to the sketch inferred from the Gene Structure Display Server (http://gsds. cbi.pku.edu.cn) by aligning the cis-elements-out DNA sequence and the corresponding full-length DNA sequence of TaNOX-pros with "ATG" and "TAA" added at 5′ and 3′ ends, respectively. The prediction score of every cis-element is greater than or equal to 5. In general, the selected cis-elements were first drawn on the sense strands instead of the antisense strands unless there were not the cis-elements on the sense strands but on the antisense strands.

Then, seven promoter sequences were cloned by PCR using the gene-specific primers, with the isolated genomic DNA from wheat leaves as the template. The promoter sequences was then cloned into the corresponding site of the dual luciferase reporter expression vector (Basic vector) instead of the 2×35S promoter in front of the Rluc gene. The Basic vector contains both expression cassettes of firefly luciferase (Fluc) and Renilla luciferase (Rluc), and the more information about it could refer to Fig. 7A and a previous study (Gu et al., 2013). Three or more monoclones were sent for sequencing to identify each promoter sequence and related vector construction (GenScript Co., Ltd, Nanjing, China). The primers used for cloning the promoters and constructing the dual luciferase reporter expression vectors were listed in Supplementary Table S6.

The preparation of rice protoplasts and the PEG-mediated transformation were performed as described previously (Zhang et al., 2011). Following transformation, the protoplasts were resuspended in 1 mL cell culture solution I per well and plated in 12-well plates (Nest Biotech Co., Ltd, Jiangsu, China) for incubation at 45 rpm at 23°C for 16 h in the dark. In order to determine the responses of the TaNOX-pros to temperature stresses and hormone treatments, the transformed protoplasts were incubated at 23°C for 2 h and then were placed at 42°C or 4°C or treated with exogenous abscisic acid (ABA) or methyl jasmonic acid (MeJA) for 14 h, respectively, while the control groups were still placed at 23°C for 14 h until harvested for luciferase activity detection.

Luciferase activity detection was performed with the Dual-Luciferase®Reporter Assay System (DLTAS) (www.promega.com) with some modification (Gu et al., 2013). The cultures from one well were put into a 1.5 ml of Eppendorf tube and then harvested by centrifugation at 12,000 rpm for 5 min. After being re-suspended in 30 μl potassium phosphate lysis buffer (PLB, provided by DLTAS kit), the protoplasts were freeze-thawed twice at -80℃ and then centrifuged at 12,000 rpm at 4℃ for 10 min. The resulted supernatants were then collected for luciferase activity detection. LSD's t-tests were performed using SPSS 16.0 software. Statistical differences between means (Rluc activity normalized against Fluc activity) from the test and control groups were considered significant at $P \leq 0.05$ based on one-way ANOVA. All values were shown as the average of the data collected from three independent experiments, each having three replicates.

Plant materials, treatments, expression profiles and coexpression network analysis

The expression patterns of wheat NOX family genes at different developmental stages and in different tissues were carried out with the online Genevestigator v3 (https://genevestigator.com/gv/). Results were

given as heat maps by submitting the probe ID of the genes into the Ta_mRNASeq_WHEAT_GL-0 database in Genevestigator v3. The inducible expression patterns of the genes were obtained in the same way, while the data from both the Ta_mRNASeq_WHEAT_GL-0 database and the TA_AFFY_WHEAT-0 database in Genevestigator v3.

To construct the coexpression patterns of wheat NOX genes with the others, the gene expression data from ten different developmental stages and/or twenty two different tissues of wheat plants were considered and the probe IDs of eight NOX genes including TaNOX2-1DL, TaNOX3-1DL, TaNOX7-3AS, TaNOX8-4DL, TaNOX12-5BL, TaNOX13-5AL, TaFRO3-2BL and TaFRO4-2BL were submitted into the Ta_mRNASeq_WHEAT_GL-0 database to search for the potential coexpression genes using the Similarity Search-Co-Expression tool in Genevestigator v3. The coexpression patterns were then constructed and presented as heat maps and also table lists.

For the analysis of the tissue-specific expression profiles of wheat NOX genes, seven tissues of wheat (*Triticum aestivum* cv. Chinese Spring) including young leaf, young root, leaf, stem, leaf sheath, young panicle and panicle were harvested to isolate the total RNA for further qRT-PCR. For analysis of the inducible expression profiles of the NOX genes, the 14 d old hydroponic seedlings that were treated with 4℃, 40℃, 200 mM NaCl, 20% PEG6000, 30 μM MV, 100 μM MeJA and 100 μM ABA respectively, for 0, 12 and 24 h, were used as the materials for RNA extraction. For the analysis of the coexpression levels of *TaNOXs* and other genes in wheat, the 7 d old seedlings treated with 42℃ for 24 h, 200 mM NaCl or 10 μM MeJA or 10 μM ABA for 6 h, soil drought for 2 d, and nature drying for 30 min respectively, were used as the materials for RNA extraction. Total RNA was extracted from the different samples using RNAiso TM Plus (Takara, Dalian, China) and treated with RNase-free DNase I (Takara, Dalian, China). The concentration and quality of the RNAs were tested by the NanoDrop 1000 Spectrophotometers (Thermo, USA) and the first-strand cDNA synthesized by using EasyScript First-Strand cDNA Synthesis SuperMix (TransGen Biotech, Beijing, China). The gene expression levels were detected with the UltraSYBR Mixture (Kangwei, Beijing, China) using a real-time PCR detection system CFX96 (Bio-Rad, Hercules, California, America) with *TaActin* (AB181991.1) and *TaGAPDH* (ABS59297.1) as the internal transcript level controls. All the expression level represent the mean ± SD of data collected three independent experiments, each having three or four replicates. The detailed primer sequences are shown in Supplementary Table S6.

Reference

1. Zhang, Y., Su, J., Duan, S., Ao, Y., Dai, J., Liu, J. et al. (2011). A highly efficient rice green tissue protoplast system for transient gene expression and studying light/chloroplast-related processes. *Plant methods*, 7, 30. doi: 10.1186/1746-4811-7-30
2. Lescot, M., Dehais, P., Thijs, G., Marchal, K., Moreau, Y., Van de Peer, Y. et al. (2002). PlantCARE, a database of plant cis-acting regulatory elements and a portal to tools for in silico analysis of promoter sequences. *Nucleic acids research*, 30, 325-327.
3. Gu, L., Han, Z., Zhang, L., Downie, B. and Zhao, T. (2013). Functional analysis of the 5' regulatory region of the maize GALACTINOL SYNTHASE2 gene. *Plant science: an international journal of experimental plant biology*, 213, 38-45. doi: 10.1016/j.plantsci.2013.09.002
